# Endothelial‐Smooth Muscle Cell Interactions in a Shear‐Exposed Intimal Hyperplasia on‐a‐Dish Model to Evaluate Therapeutic Strategies

**DOI:** 10.1002/advs.202202317

**Published:** 2022-08-15

**Authors:** Andreia Fernandes, Arnaud Miéville, Franziska Grob, Tadahiro Yamashita, Julia Mehl, Vahid Hosseini, Maximilian Y. Emmert, Volkmar Falk, Viola Vogel

**Affiliations:** ^1^ Laboratory of Applied Mechanobiology Institute of Translational Medicine Department of Health Sciences and Technology ETH Zurich 8093 Zurich Switzerland; ^2^ Department of Cardiovascular Surgery Charité Universitätsmedizin Berlin 10117 Berlin Germany; ^3^ Department of Cardiothoracic and Vascular Surgery German Heart Center Berlin 13353 Berlin Germany; ^4^ Institute for Regenerative Medicine (IREM) University of Zurich 8006 Zurich Switzerland; ^5^ Department of Health Sciences and Technology ETH Zurich 8093 Zurich Switzerland; ^6^ Present address: Department of System Design Engineering Keio University 108‐8345 Yokohama Japan; ^7^ Present address: Julius Wolff Institute Berlin Institute of Health Charité Universitätsmedizin Berlin 10117 Berlin Germany

**Keywords:** drug screening, endothelial cell networks, flow, in vitro coculture model, vascular injury

## Abstract

Intimal hyperplasia (IH) represents a major challenge following cardiovascular interventions. While mechanisms are poorly understood, the inefficient preventive methods incentivize the search for novel therapies. A vessel‐on‐a‐dish platform is presented, consisting of direct‐contact cocultures with human primary endothelial cells (ECs) and smooth muscle cells (SMCs) exposed to both laminar pulsatile and disturbed flow on an orbital shaker. With contractile SMCs sitting below a confluent EC layer, a model that successfully replicates the architecture of a quiescent vessel wall is created. In the novel IH model, ECs are seeded on synthetic SMCs at low density, mimicking reendothelization after vascular injury. Over 3 days of coculture, ECs transition from a network conformation to confluent 2D islands, as promoted by pulsatile flow, resulting in a “defected” EC monolayer. In defected regions, SMCs incorporated plasma fibronectin into fibers, increased proliferation, and formed multilayers, similarly to IH in vivo. These phenomena are inhibited under confluent EC layers, supporting therapeutic approaches that focus on endothelial regeneration rather than inhibiting proliferation, as illustrated in a proof‐of‐concept experiment with Paclitaxel. Thus, this in vitro system offers a new tool to study EC‐SMC communication in IH pathophysiology, while providing an easy‐to‐use translational disease model platform for low‐cost and high‐content therapeutic development.

## Graphical abstract

A vessel‐on‐a‐dish platform is introduced, which allows to explore both healthy vasculature and intimal hyperplasia models. It consists of direct‐contact cocultures of endothelial cells (EC) and smooth muscle cells (SMC) exposed to shear on an orbital shaker and is well suited to investigate EC‐SMC interactions, as well as for drug screening applications for preventing intimal hyperplasia in stents and grafts after vascular injury.

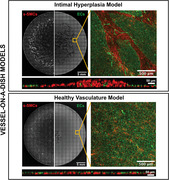

## 1 Introduction

Intimal hyperplasia (IH) induced stenosis remains a major clinical problem that affects the long‐term outcome of stents and vascular conduits.^[^ ^1^, ^2^, ^3^ ^]^ IH is a remodeling of the vessel wall that results in the thickening of the intimal layer, eventually leading to stenosis. Although the use of drug‐eluting stents has significantly reduced the incidence of in‐stent restenosis by 50–70%, still a 3–20% rate is reported depending on the type of drug, the stent material, complexity of the vascular lesion, and the duration of follow‐up.^[^ ^4^, ^5^ ^]^ The saphenous vein, which is commonly used as a conduit in coronary artery bypass grafting (CABG), is associated with 40%‐50% of late graft failure.^[^ ^6^ ^]^ Despite the recent advances of synthetic and tissue engineered grafts, an ideal alternative other than native arterial grafts is not yet available.^[^ ^7^ ^]^

Both stented vascular segments and vascular conduits are subject to vascular injury, which is an established trigger for IH. Stents are applied using high pressure balloons to dilate atherosclerotic lesions and cause local vascular injury.^[^ ^8^ ^]^ Vascular conduits such as saphenous vein grafts may be injured during harvest by mechanical manipulation, ischemia‐reperfusion injury and long‐term due to chronic arterialization.^[^ ^6^ ^]^ Endothelial injury triggers platelet activation and thrombus formation and the recruitment of inflammatory cells, which in turn activate endothelial cells (ECs) into a proliferative and proinflammatory state, affecting their function and communication with subjacent vascular smooth muscle cells (SMCs).^[^ ^9^ ^]^ Without the endothelium as a protective barrier, SMCs become exposed to shear and blood circulating factors that trigger their phenotypical modulation from contractile to synthetic.^[^ ^10^, ^11^ ^]^ This phenotypic shift is correlated with the lower expression of contractile markers ^[^ ^10^ ^]^ such as alpha smooth muscle actin (*α*SMA), an actin isoform which incorporates into stress fibers,^[^ ^12^ ^]^ and Calponin, a filament‐associated contractility regulator protein and inhibitor of actin‐activated myosin ATPase.^[^ ^13^ ^]^ The only reliable marker of synthetic SMCs is S100A4, a small calcium‐binding protein involved in cell proliferation and migration that inhibits the phosphorylation of target proteins.^[^ ^14^ ^]^ However, since it was only identified in 2007, the downregulated expression of contractile markers is still the most common characterization of the synthetic phenotype. Synthetic SMCs have increased ability to proliferate, migrate and synthesize ECM proteins, which ultimately leads to IH.^[^ ^15^ ^]^

IH is the result of a complex interplay between immune cells, platelets, ECs, and SMCs, which is further regulated by blood flow.^[^ ^16^, ^17^ ^]^ Current therapies mostly focus on anti‐mitotic drugs to control SMC proliferation, since IH ultimately results from SMC proliferation and migration towards the intima.^[^ ^18^, ^19^ ^]^ The first commercially available drug‐eluting stent, which was launched in 2002, gradually releases either sirolimus (antiproliferative and immunosuppressive) or paclitaxel (antiproliferative) from a nondegradable polymer coating.^[^ ^3^ ^]^ Since such drug‐eluting stents have been associated with higher risk of mortality, due to late in‐stent‐thrombosis related to delayed reendothelialization, the quest for improved drug‐eluting stent technologies continues.^[^ ^20^, ^21^, ^22^ ^]^

The current strategies to study IH and to test new drug targets are mainly based on animal models, which are usually expensive, logistically challenging, ethically questionable, and limited in translation due to interspecies differences.^[^ ^23^ ^]^ Since replicating physiological conditions in vitro is challenging, scientists have taken a reductionist approach to mimic and study the underlying mechanisms of diseases.^[^ ^24^, ^25^ ^]^ While such in vitro platforms for disease modeling and drug testing may represent highly valuable tools for therapeutic development, they also need to be clinically relevant and therefore cannot be too simplistic. For instance, denudation and activation of the endothelium after vascular injury is also a very crucial trigger of IH and should therefore not be neglected.^[^ ^9^ ^]^ Next, shear forces as imposed by blood flow are known to be important regulators of endothelial cell function^[^ ^26^ ^]^ and to induce SMC shift towards a synthetic phenotype by reducing mRNA expression of *α*SMA and Calponin and by increasing proliferation.^[^ ^27^ ^]^ Hence, it is important to study these interactions under hemodynamic conditions. Moreover, despite the basement membrane in between, ECs have physical contact with SMCs in the native tissue and communicate through Notch3‐Jagged1, Connexins, and Ephrins.^[^ ^28^ ^]^ Additionally, ECs and SMCs interact via growth factors, miRNAs, extracellular vesicles, and ECM.^[^ ^28^, ^29^, ^30^ ^]^ Several in vitro models are available to study EC‐SMC communication in both static and shear conditions.^[^ ^31^ ^]^ However, these studies have been carried out with different cell sources, with or without direct contact, from 2D to 3D environments, different hemodynamic conditions, and often neglecting the SMC phenotype. This large variety of heterogeneous studies has led to contradictory results and lack of reproducibility. Further, only few of these models are brought into context of IH.^[^ ^32^, ^33^, ^34^ ^]^

Since the multiple factors discussed above are acting synergistically, we created a high‐content platform to mimic not only the sandwich architecture of the arterial wall, but also endothelial injury, with the goal to address the existing knowledge gaps and to place our findings in the context of IH. ECs were seeded at confluent or sub‐confluent density on top of either synthetic (s‐SMC) or contractile SMCs (c‐SMC), allowing for direct contact. Certain blood flow characteristics were mimicked, by exposing the cocultures to shear for 3 days using a 6‐well plate on an orbital shaker. This platform enables a direct comparison of different flow conditions within the same sample, which would not be possible using traditional transwell assays.^[^ ^35^, ^36^, ^37^, ^38^ ^]^ At the periphery of the well, cells are exposed to laminar and high‐velocity pulsatile flow that mimics physiological conditions, while the cells in the center sense low‐velocity disturbed flow which characterizes atheroprone regions in vivo (Figure S1A, Supporting Information).^[^ ^39^, ^40^, ^41^ ^]^ Fluid dynamics in this system has been well characterized by computer simulations.^[^ ^42^, ^43^, ^44^ ^]^

With this vessel‐on‐a‐dish system, we explored not only a model of quiescent vasculature that mimics the sandwich architecture of the vessel wall, but also an IH model mimicking partial endothelial denudation and intimal thickening. The comparison between these different models in this easy‐to‐use platform provides crucial insights into the interplay between ECs and SMCs at the cellular and tissue level under hemodynamic conditions and into the context of vascular injury and IH. Finally, we demonstrate that this platform is suitable for drug screening in a proof‐of‐concept study using paclitaxel.

## 2 Results

### 2.1 Development of Protocols to Create Models of Vascular Health and Disease

**Figure 1:**
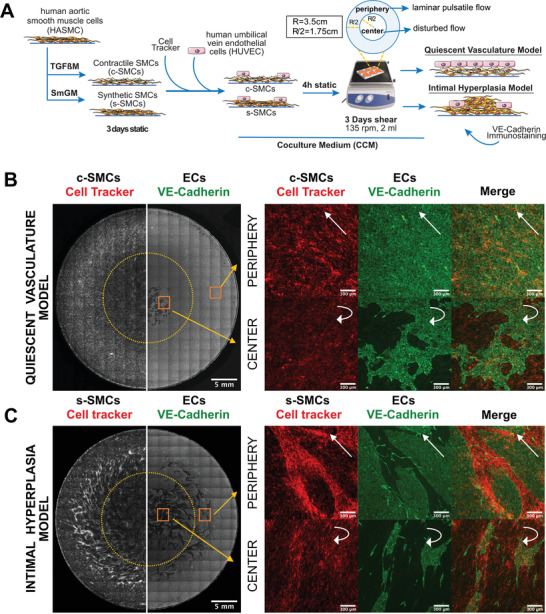
EC cocultures with either c‐SMC or s‐SMC to mimic quiescent vasculature and IH, respectively. A) Schematics of the method to create quiescent vasculature and intimal hyperplasia models. TG*β*M or SmGM were used to induce either SMC contractile or synthetic phenotype, as shown in Figure S2 (Supporting Information). SMCs were stained with cell tracker. ECs were seeded on top of SMCs (8 × 10^4^ cells cm^−2^) and, after 4 hours, cocultures were placed on an orbital shaker. After 3 days of shear exposure, rotating at 135 rpm with 2 mL of culture medium, samples were fixed for immunostaining. Blown out schematics of one well to define radius (R), periphery (blue) and center (white) regions, together with the type of flow in each region. Resulting models of B) quiescent vasculature and C) intimal hyperplasia , where the black/white images acquired by widefield fluorescence microscopy show the whole well as a large stitched mosaic, with SMCs shown stained with cell tracker on the left half and ECs stained with VE‐cadherin immunostaining on the right half. The rectangular grid patterns result from stitching. A punctuated yellow line separates what we define throughout as “center” and “periphery” of the well. Scale bar 5 mm. On the right side, zoomed‐in images of yellow squares show SMCs (red), ECs (green) and merged images, from the periphery and center of the well. White straight arrows show the direction of flow, while curved arrows represent disturbed flow. Scale bar 300 µm. Supporting videos of high‐resolution Z‐stacks acquired by confocal microscopy at the center and periphery of the well (in a different sample) are shown in Video S2 (Supporting Information).

Human umbilical vein endothelial cells (HUVECs) and human aortic smooth muscle cells (HASMC) have been used throughout this study and called ECs and SMCs for simplification. To create an in vitro model that recapitulates some essential features of IH versus quiescent vasculature, we selected a coculture medium (CCM, **Table** **1**) adequate for both cell types, containing 2% FBS as well as EGF and FGFb, two growth factors that are known to induce SMC migration and proliferation^[^ ^10^, ^45^, ^46^ ^]^ and to be essential for EC growth and survival.^[^ ^47^ ^]^ Since ECs are known to sense fluid flow and align parallel to it in physiological conditions,^[^ ^48^ ^]^ cells were exposed to pulsatile flow conditions in a 6‐well plate on an orbital shaker ^[^ ^41^ ^]^ at 135 rpm using 2 mL of CCM (Figure S1 and Video S1, Supporting Information). The average value of the mean wall shear stress (WSS) in human umbilical vein and aorta, from where ECs and SMCs were isolated, has been previously calculated as approximately 0.52 Pa and 0.5–1 Pa, respectively.^[^ ^49^, ^50^ ^]^ Published hydrodynamic simulations of the mean WSS on the orbital shaker for 100, 120,^[^ ^43^ ^]^ and 200 rpm^[^ ^44^ ^]^ are summarized in Figure S1A (Supporting Information). Based on these simulations, we estimated that with our setup (135 rpm) mean WSS ranged from 0.4 Pa to 1.2 Pa in the peripheral region of the well and is therefore within the physiological range, while flow is disturbed and multidirectional in the central region, with an estimated mean WSS between 0.3 Pa and 0.8 Pa (Figure S1A, Supporting Information). Furthermore, considering that the frequency of shaker rotation can be correlated to the heartbeat rate, the 135 rpm used here falls within the physiological range of an adult during exercise.^[^ ^51^ ^]^ Despite having a gradient of WSS proportional to the radial position, we segmented the image at the middle of the radial position to distinguish central and peripheral region and facilitate analysis and comparisons (**Figure** **1**A), similarly to previous studies.^[^ ^43^ ^]^

**Table 1:** Cell culture media and their respective supplements

Medium name	Basal medium	Serum	Growth factors	Supplements
Contractile phenotype inducing medium (TGFßM)	MCDB131	10% FBS	TGFß	Glutamax
Synthetic phenotype inducing medium (PDGFM)	MCDB131	10% FBS	PDGF	Glutamax
Control medium (CtrlM)	MCDB131	10% FBS	–	Glutamax
Smooth muscle growth medium (SmGM)	SmBM	5% FBS	EGF, FGFb	Insulin, Amphotericin and Gentamycin
Endothelial growth medium (EGM‐2)	EBM‐2	2% FBS	EGF, FGFb, VEGF, IGF, Heparin	Hydrocortisone, Ascorbic Acid, GA‐1000
Endothelial growth medium (m200)	m200	2% FBS	EGF, FGFb	Hydrocortisone, Amphotericin, and Gentamycin
Coculture medium (CCM)	MCDB131	2% FBS	EGF, FGFb	Glutamax, Hydrocortisone, Amphotericin, and Gentamycin
Coculture medium without growth factors (CCM‐EGF‐FGFb)	MCDB131	2% FBS	–	Glutamax, Insulin‐transferrin‐selenium

In line with the literature, ECs aligned in the direction of the flow in the peripheral region after being exposed to shear for 3 days (Figure S1B–D, Supporting Information), as seen through VE‐cadherin staining, a transmembrane protein that belongs to the mechanoreceptor complex of adherent junctions (Figure S1C, Supporting Information).^[^ ^52^, ^53^ ^]^ This proves that the cells are healthy under the selected shear parameters. On the other hand, cell alignment was random in the center of the well and monolayer was often defected (Figure S1C,D, Supporting Information). This agrees with previous studies with orbital shakers showing that ECs are more senescent, apoptotic and permeable in the center, and express more pro‐inflammatory and less homeostatic genes than ECs at the periphery.^[^ ^41^, ^54^ ^]^

Since the SMC phenotypic shift is an important trigger of IH, yet too often neglected in the literature, we developed protocols to study cocultures with both contractile or synthetic SMCs in our vessel on‐a‐dish models. To induce the synthetic phenotype, SMCs were cultured for 3 days in static conditions with smooth muscle growth medium (SmGM), which is rich in EGF and FGFb like CCM (Table 1). As expected, SMCs expressed low levels of the contractile phenotype markers *α*SMA and calponin, and high levels of the synthetic marker S100A4 (Figure S2A–C, Supporting Information). Serum‐starved medium has often been used as an inducer of SMC contractile phenotype,^[^ ^10^, ^55^, ^56^, ^57^ ^]^ which is not only physiologically irrelevant but may also potentially affect various cellular functions. Therefore, TGFß was used at a concentration that has been previously reported to induce the contractile phenotype and reduce proliferation in SMCs.^[^ ^10^, ^58^, ^59^, ^60^ ^]^ Thus, SMCs were cultured in a medium containing 10% serum and 10 ng mL^−1^ TGFß (TGFßM, Table 1), resulting in flat and large cell morphology, low expression levels of S100A4 and high levels of calponin and *α*SMA‐rich stress fibers (Figure S2A–C, Supporting Information).^[^ ^10^, ^58^ ^]^ Additionally, the expression of phenotype markers in other culture media was tested by immunostaining and western blotting (Figure S2A,C, Supporting Information).

Since EGF, FGFb, and direct exposure to shear are each known to induce the SMC synthetic phenotype,^[^ ^10^, ^61^ ^]^ we verified that the chosen coculture medium (CCM, Table 1) in combination with flow exposure on the orbital shaker did not cause the c‐SMCs to convert back to s‐SMCs (Figure S2D, Supporting Information). After placing TGFß‐induced c‐SMCs on the orbital shaker and culturing them in CCM for 3 day, which is the time frame of all our experiments, calponin and S100A4 protein levels remained unaltered, as quantified by western blot. Further, although *α*SMA levels reduced slightly, they remained several folds higher than in SmGM‐induced s‐SMCs after exposure to shear in CCM, which further motivated us to proceed to coculture experiments using these experimental parameters.

Finally, a preliminary fibronectin ECM was shown to be assembled by SMCs in both TGFßM and SmGM, as shown by immunofluorescence (Figure S2E,F, Supporting Information). However, fibronectin ECM was significantly more abundant in TGFßM compared to SmGM, not only because TGFß is known to induce fibronectin synthesis, but also because TGFßM has a higher concentration of FBS and therefore more soluble fibronectin available. ^[^ ^62^, ^63^ ^]^ In fact, fibronectin matrix was similarly abundant in CtrlM (Figure S2E, Supporting Information), which has the same FBS concentration but no TGFß (Table 1).

Based on these results, the decision was made to use SmGM and TGFßM as s‐SMC and c‐SMC phenotype inducers respectively, 135 rpm shaker rotation speed for a physiological WSS range, and CCM as a coculture medium throughout the whole study.

### 2.2 On Top of c‐SMCs, ECs Form a Confluent Monolayer and Align with the Flow

Aiming to recapitulate central features of a quiescent vessel wall, ECs were cultured on top of a layer of TGFß‐induced c‐SMCs labeled with cell tracker and exposed to flow on the orbital shaker for 3 days in CCM (Figure 1A). To ensure that cells were not depleted of essential nutrients, while allowing for the exchange of secreted paracrine factors that steer SMC‐EC communication, only half of culture medium was collected every 24 hours and substituted by fresh medium. The same was done for all samples throughout the entire study. During the first 2 days on the orbital shaker, about 26% of ECs proliferated on c‐SMCs at the periphery of the well, dropping to 6% on day 3 (Figure S3B, Supporting Information). After 3 days, the EC layer was confluent, with only few defects in the center of the well where the flow was disturbed (Figure 1B and Figure S3A, Supporting Information). In monocultures, proliferating ECs covered approximately 50% at the periphery on day 1, and peripheral confluency was reached already after 48 hours (Figures S3 and S4, Supporting Information). As expected, in both mono‐ and cocultures, ECs aligned parallel to the flow (Figure 1B and Figures S1C,D and S4, Supporting Information). c‐SMCs in coculture were stained for *α*SMA, calponin and S100A4, confirming that the expression of these markers remained relatively stable (Figure S5, Supporting Information), compared to the start of coculture (Figure S2A, Supporting Information). Taken together, this sandwich coculture not only reproduced published data,^[^ ^59^, ^64^ ^]^ but also recapitulated central features of a quiescent endothelium at day 3.^[^ ^65^ ^]^ Hence, it may not only serve as a good control for the IH model, but also offer a protocol to produce large tissue areas for vascular grafts or coating of implanted medical devices.

### 2.3 On Top of s‐SMCs, ECs Form Networks That Grow into Confluent 2D Islands Over Time

**Figure 2:**
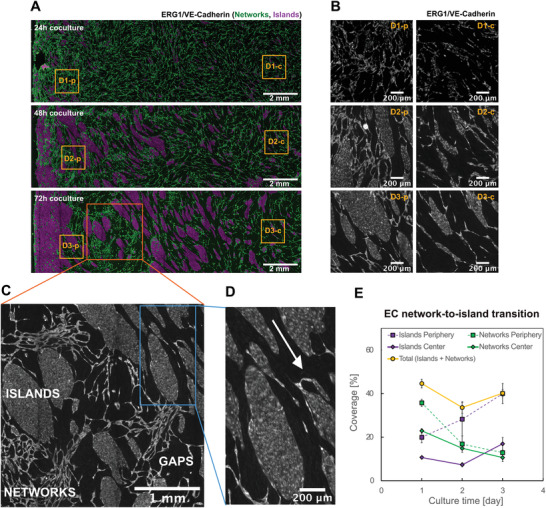
ECs initially form networks on top of s‐SMCs (IH model) and grow into confluent 2D islands over time. A) Output image from texture analysis distinguishes EC networks (green) from EC islands (purple). Images show a segment of the coculture from the periphery to center of the well at 24, 48, and 72 h. The original images acquired by widefield fluorescence microscopy show ERG1 and VE‐cadherin double immunostaining using the same secondary antibody. s‐SMCs are present, but not shown here for better visualization. Scale bar 2 mm. B) Zoomed‐in images of regions marked with yellow box in (A), showing original ERG1/VE‐cadherin staining. D = Day, p = periphery, c = center. Scale bar 200 µm. C) Zoomed‐in image of region marked with an orange box in (A), showing original ERG1/VE‐cadherin staining, and defining EC island, EC networks and gap regions. Scale bar 1 mm. D) Zoomed‐in image of region marked with a blue box in (C), showing original ERG1/VE‐cadherin staining and ECs aligning with the flow. White arrow shows the flow direction. Scale bar 200 µm. E) Quantification of percentage of area covered with EC networks (green), EC islands (purple) in center and periphery regions of the well, and the total percentage of area covered with ECs in the whole well (yellow). Analysis was done using different regions of interest from the images in Figure (A), therefore showing local variance.

**Figure 3:**
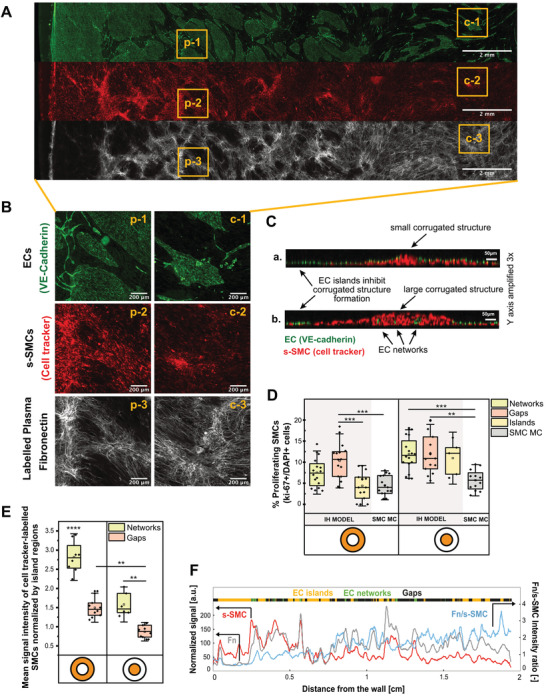
In the IH model, in regions of EC layer defects, s‐SMCs increased proliferation, formed pronounced corrugated structures, and assembled plasma fibronectin fibers. A) Widefield fluorescence microscope image of IH model, showing a segment of the coculture from periphery to center of the well. The three channels are shown separately: VE‐cadherin immunostaining (green), cell tracker labelled s‐SMCs (red), supplemented plasma‐fibronectin labeled with Alexa 647 that got incorporated into the ECM (gray). Scale bar 2 mm. B) Zoomed‐in images of yellow squares. p = periphery; c = center. Scale bar 200 µm. C) Orthogonal views of z‐stacks taken with a confocal microscope, showing a small (a) and a large (b) corrugated structure in the peripheral region of the well with EC networks inside. ECs, immunostained with VE‐cadherin (green) and cell tracker labelled SMCs (red). Scale bar XY axis 50 µm. Scale bar XZ axis 150 µm (Y axis amplified 3×). Videos of z‐stacks used to create these orthogonal views are shown in Video S3 (Supporting Information). D) Graph showing the percentage of proliferative SMCs at 3 days of coculture in different regions of the tissue (EC islands, gaps, and EC networks, as defined in Figure 2C) in both center and periphery regions of the well. IH model is compared with s‐SMC monocultures (SMC MC). Total number of nuclei was calculated through DAPI stain. ERG1 immunostaining was used to distinguish EC nuclei from SMC nuclei and ki‐67 to identify proliferative cells. For statistical analysis, one‐way ANOVA followed by Tukey's test was performed (**** *p* < 0.001, *** *p* < 0.01, ** *p* <0.1, *p < 0.5). E) Graph showing mean intensity of the SMC channel (cell tracker) in different regions of the tissue (gaps and networks), in both center and periphery regions of the well, normalized by cell tracker intensity on regions of EC islands. Since the cell tracker intensity is approximately the same per cell, the intensity is directly proportional to the number of cells. For statistical analysis, one‐way ANOVA followed by Tukey's test was performed (**** *p* < 0.001, *** *p* < 0.01, ** *p* < 0.1, *p < 0.5). F) Graph showing intensity plots of cell tracker (s‐SMCs, red) and labeled plasma fibronectin (Fn, gray), binarized signal of VE‐cadherin immunostaining (EC islands— yellow, EC networks—green, Gaps—black) and intensity of plasma fibronectin signal normalized by the intensity of cell tracker (Fn/s‐SMC, blue) from periphery to center of the well. s‐SMC and Fn signals are normalized to a 0–255 scale.

In contrast to cocultures with c‐SMCs, when ECs were seeded with the same density (8 × 10^4^ cells cm^−2^) on top of a s‐SMC layer, using the same protocol (Figure 1A), the endothelium did not reach confluency in all peripheral areas after 3 days on the orbital shaker (Figures 1C, **2**, and **3**A,B and Figure S3A, Supporting Information). Since the only difference between these two models was the phenotype of SMCs, this suggests that EC layers grow more slowly towards confluency on top of s‐SMC compared to c‐SMCs, as reported before.^[^ ^59^, ^66^, ^67^ ^]^ In fact, EC proliferation was significantly lower at the periphery in the first 48 h of coculture with s‐SMCs (mean 15%), as compared to the quiescent model (mean 26%) (Figure S3B, Supporting Information).

To investigate the evolution of the endothelium over time, we compared independently grown tissues fixed at different time points, since live‐cell imaging was not applicable for a sample under motion on the orbital shaker. Interestingly, ECs self‐assembled into networks on top of s‐SMCs after 24h of coculture (Figure 2A,B), a morphogenesis that was previously observed in EC monocultures on compliant surfaces,^[^ ^68^ ^]^ in collagen and fibrin gels,^[^ ^69^ ^]^ as well as in cocultures with SMCs,^[^ ^70^, ^71^ ^]^ osteoblasts,^[^ ^72^ ^]^ or fibroblasts.^[^ ^73^ ^]^ Only after 48 h, ECs started to form islands of confluent endothelium with the typical cobblestone morphology, especially at the periphery of the well. Finally, after 72 h, ECs formed a heterogeneous layer composed of EC networks and different sized 2D islands of confluent endothelium containing flow‐aligned ECs (Figures 1C, 2, and 3A and Figure S6, Supporting Information). The size of these islands decreased from the periphery to the center of the well, reflecting the effects of the shear gradient and flow profile across the well (Figures 2A and 3A). At day 3, the EC networks were generally denser at the periphery of the well (Figure 2A,B), but the overall abundance and density were different between experiments (Figures 1C, 2A, and 3A). Moreover, the presence of networks was independent of any individual component in the culture medium, but was reduced with the removal of both EGF and FGFb from the culture medium (Figure S7, Supporting Information).

Overall, the area covered with EC networks decreased, while the area with 2D islands increased over time (Figure 2E). This transition seemed to be mostly driven by cellular migration and morphological changes, since ECs proliferated significantly less on top of s‐SMCs in the first 24 hours as compared to cocultures with c‐SMCs and monocultures, and the number of proliferating cells was still less than 10% at day 3 (Figure S3B, Supporting Information). Further, the total EC coverage stayed at a similar level throughout the 3 days (Figure 2E), despite the drastic change in the morphology of the covering ECs.

To verify whether this morphogenic shift from EC networks towards confluent islands was dependent on EC density, the double number of ECs was seeded on top of s‐SMCs (Figures S8 and S9, Supporting Information). The same network‐to‐island transition was observed, but in the high‐density model a dense mix of coexisting large islands and networks was observed already after 24 hours (Figure S10, Supporting Information). After 48 hours, very few EC networks remained, and the EC layer was almost confluent. At day 3, the EC layer was confluent at the periphery with few gaps in the center, and the number of proliferating cells was very low, similarly to the quiescent model (Figures S3B and S10, Supporting Information).

Taken together, our results suggest that ECs preferentially form networks when at low density on s‐SMCs, and the familiar 2D monolayer at high density. Thus, this model not only replicates characteristics of a defected endothelium but also may help us better understand how it regenerates in vivo after vascular injury under a range of fluid shear.

### 2.4 Corrugated Structures Composed of s‐SMC Multilayers Form in Cocultures with a Defected EC Layer under Shear

As a result of the crosstalk with a defected EC monolayer, s‐SMCs formed corrugated structures resulting in a hill‐valley topography that could be observed in a large mosaic after tile stitching (Figures 1C and 3A and Figure S11, Supporting Information). The cell tracker intensity peaks correspond to the hills of these corrugated structures, resulted from the thickening of the s‐SMC layer and consisted of 2–3 s‐SMCs on top of each other (Figure 3B,C **;** Videos S2–S4, Supporting Information). These corrugations are much more pronounced and defined as compared to the typical hill‐valley topography observed in SMC monocultures under shear (Figures S11 and S12, Supporting Information).

Interestingly, corrugated structures colocalized with regions of EC layer “gaps” and “networks” (Figure 3F). But despite corrugations only forming in the defected areas of the EC layer, a defect did not necessarily result in a corrugated structure (Figure 3F; Video S2, Supporting Information). Since the dimensions of corrugated structures was highly dependent on the size of EC layer defects, their diameter and length varied a lot within the tissue and between experiments, as shown in some examples in Figure S11 (Supporting Information). Imagining that our in vitro models represent a vessel wall cut open like a book, the observed thickening of s‐SMC layer and growth to fill the gaps of the defected EC layer show characteristic hallmarks of IH in vivo.

These corrugations are robustly formed in different coculture media, such as m200 (Invitrogen) and EGM‐2 (Lonza) (Figure S13, Supporting Information), and in wells where the center was made non‐adherent to cells with a thin PEG gel coating, proving that the formation of corrugated structures was not caused by the secretion of paracrine factors from cells in the disturbed flow region (Figure S14, Supporting Information). On the other hand, formation of pronounced corrugated structures was inhibited when both EGF and FGFb were removed from the culture medium, even in the presence of a defected endothelium (Figure S15, Supporting Information). Furthermore, areas of dense EC networks typically colocalized with regions of very pronounced thickening of the s‐SMC layer (Figure 3E), and Z‐stack images of these regions show that the EC networks can be entrapped inside the corrugated structures (Figure 3C‐b; Videos S2A and S3B, Supporting Information). However, corrugated structures were also found in EC gaps without any networks, which suggests that networks are not necessary to induce their formation (Figure 3B,C‐a, and Figure S16, Supporting Information).

### 2.5 Proinflammatory Cytokines Are Secreted by ECs and SMCs in Coculture

Since IH is characterized by an increase of inflammatory cytokines at the site of injury, but our model does not include immune players, the concentration of the most relevant cytokines was analyzed in the supernatants using an array (Figure S17, Supporting Information). In all cocultures and monocultures IL‐8, MCP1, TIMP‐1 and TIMP‐2 are present at high concentrations. Further, more pro‐inflammatory cytokines were detected in the supernatant in coculture with s‐SMCs as compared to EC monocultures (e.g., ENA‐78, Gro, Gro‐alpha, and IL‐6, IL‐8). This is highly interesting, as it shows that the cell niche environment created in our in vitro model system induces a slight proinflammatory response as mediated by ECs‐SMC interactions, despite the absence of immune cells. Note that this assay does not allow to distinguish whether the cytokines are secreted by cells in the region of disturbed flow (center) or laminar flow (periphery). Secreted factors from one region of the well can affect the secretome from the other.

### 2.6 Confluent EC Islands Stop the Formation of Corrugated Structures by Inhibiting Proliferation of Subjacent s‐SMCs and Inducing *α*‐SMA Expression

Since images show that corrugated structures did not form below EC islands (Figures 1C and 3A–C, and Figure S16, Supporting Information), the inhibitory role of confluent ECs on SMC proliferation was investigated. Thus, all nuclei were stained with DAPI, proliferative cells with the nuclear marker ki‐67 and EC nuclei with ERG1. s‐SMC nuclei were DAPI positive and ERG1 negative, which allowed us to calculate the number of proliferative cells in different selected areas of the tissues. Indeed, while the number of proliferative s‐SMCs significantly increased in gaps and regions with EC networks, below EC islands proliferation remained as low as in SMC monocultures (Figure 3D). This suggests that corrugated structures resulted from increased s‐SMC proliferation induced by nearby ECs, without excluding the possibility that s‐SMC might also have migrated. This increase of proliferation was dependent on the presence of EGF and FGFb in the culture medium (Figure S18, Supporting Information). Moreover, the fact that in s‐SMC monocultures there was no significant difference in proliferation between center and periphery of the well, demonstrates that shear had no direct effect on the formation of the corrugated structures (Figure 3D).

**Figure 4:**
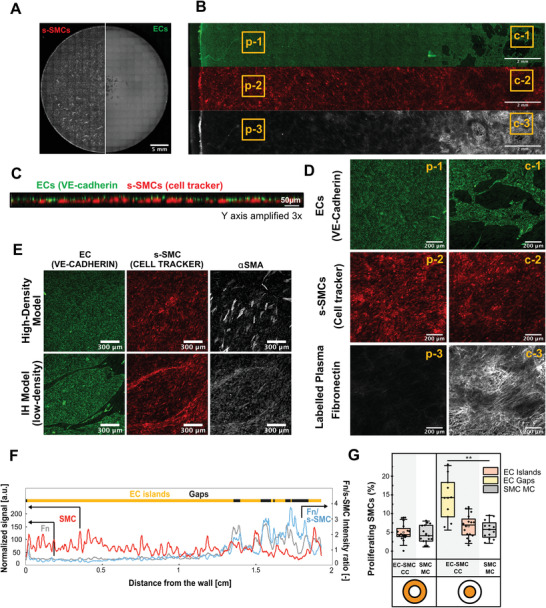
Confluent EC monolayer inhibits formation of s‐SMC corrugated structures and incorporation of plasma fibronectin in ECM, while enhancing expression of *α*SMA. A‐F) Co‐culture where ECs were seeded at high density on s‐SMCs (high‐density model). A) Black and white image, acquired by widefield fluorescence microscopy, show the whole well as a large stitched mosaic, with SMCs stained with cell tracker on the left half and ECs stained with VE‐cadherin immunostaining on the right half. The rectangular grid patterns result from stitching. Scale bar 5 mm. Videos of high‐resolution Z‐stacks acquired by confocal microscopy at the center and periphery of the well are shown in Video S5 (Supporting Information). B) Widefield fluorescent image, showing a fraction of the tissue from border to center of the well. The three channels are shown separately: VE‐cadherin immunostaining (green), cell tracker in s‐SMCs (red), supplemented plasma‐fibronectin labeled with Alexa 647 (gray). Scale bar 2 mm. C) Orthogonal view of a z‐stack taken with a confocal microscope, showing a layer of ECs on top of a layer of s‐SMCs, at the periphery of the well. ECs, immunostained with VE‐cadherin (green) and cell tracker labeled SMCs (red). Scale bar XY axis 50 µm. Scale bar XZ axis 150 µm (Y axis amplified 3×). Video of the z‐stack used to create this orthogonal view is shown in Video S6 (Supporting Information). D) Zoomed‐in images of yellow squares in (B). p = periphery, c = center. Scale bar 200 µm. E) Confocal images taken from the periphery, show *α*SMA expression in SMCs under EC monolayer. VE‐cadherin immunostaining (green), cell tracker stained SMCs (red), *α*SMA immunostaining (grey). Scale bar 200 µm. F) Graph showing intensity plot of cell tracker (s‐SMCs, red) and labeled plasma fibronectin (Fn, gray), binarized signal of VE‐cadherin (EC islands ‐ yellow, Gaps ‐ black) and intensity of plasma fibronectin signal normalized by the intensity of cell tracker (Fn/s‐SMC, blue) from periphery to center of the well. s‐SMC and Fn signals are normalized to a 0–255 scale. G) Graph showing the percentage of proliferating SMCs at 3 days of coculture in different regions of the tissue (EC islands and gaps, as defined in Figure 2C) in both center and periphery of the well. High‐density model (EC‐SMC CC) is compared with s‐SMC monocultures (SMC MC). Total number of nuclei was calculated through DAPI stain. ERG1 immunostaining was used to distinguish EC nuclei from SMC nuclei and ki‐67 to identify proliferative cells. For statistical analysis, one‐way ANOVA followed by Tukey's test was performed (**** *p* < 0.001, *** *p* < 0.01, ** *p* < 0.1, *p < 0.5).

Since proliferation was the lowest in EC island regions (Figure 3D), our results indicate that a confluent EC layer inhibits the formation of corrugated structures by maintaining s‐SMC proliferation low. To confirm this hypothesis, ECs were seeded at high density (i.e., double number of cells) on top of s‐SMCs (Figures S8 and S9, Supporting Information) and cultured on the orbital shaker. After 3 days, the endothelium was confluent at the periphery (**Figure** **4**A,B,D, and Figure S10, Supporting Information) with stable adherent junctions and ECs aligning in the direction of the flow (Figure 4D and Figures S6 and S10B and Videos S5A and S7, Supporting Information). However, the EC layer still had small defects in the center of the well where the flow was disturbed (Figure 4A,B,D, Figure S10, and Video S5B, Supporting Information). As expected, s‐SMC proliferation in high‐density cocultures was as low as in SMC monocultures, except in the gap regions in the center (Figure 4G), and the formation of corrugated structure was prevented (Figure 4A–D,F).

Moreover, SMCs also increased the number of cells expressing fiber‐associated‐*α*SMA in the high‐density model, as seen by immunostaining (Figure 4E). This result confirms previous work showing that ECs induce the contractile SMC phenotype in static cocultures with a transwell assay.^[^ ^34^, ^74^ ^]^ Since ECs forming stable VE‐cadherin cell–cell contacts have never been reported to induce expression of fiber‐associated *α*SMA in SMCs, we are confident that the observed *α*SMA‐expressing cells are SMCs that underwent a phenotypic shift. Further, since the number of *α*SMA positive cells was very low in SMC monocultures exposed to flow and low‐density cocultures (Figure S5, Supporting Information), our data suggest that this phenotypic switch is only induced by direct contact with a confluent EC layer, while SMCs are protected from direct exposure to shear.

### 2.7 Supplemented Plasma Fibronectin Is Incorporated by SMCs into the ECM, but Only in Regions Where the Endothelium Is Defected

After endothelial denudation, blood circulating plasma fibronectin becomes accessible and can be incorporated by SMCs into their native fibronectin ECM fibers, which together with cell‐secreted fibronectin serve as template for the assembly of other ECM proteins, such as collagen I.^[^ ^75^, ^76^ ^]^ Since later stages of IH are characterized by increased ECM biosynthesis,^[^ ^17^ ^]^ we investigated whether ECM assembly was also increased in our IH model. Thus, to replicate exposure to blood circulating fibronectin, fluorescently labeled human plasma fibronectin was added to the supernatant during the 3 days of coculture. As expected, supplemented labeled fibronectin was incorporated into the ECM and assembled into fibers, most prominently in locations where the endothelium was defected (Figure 3A**–**B,F). The correlation between s‐SMC and plasma fibronectin was high, suggesting that s‐SMCs are mostly responsible for the incorporation of plasma fibronectin into their newly synthesized ECM (Figure 3F). Orientation of fibronectin fibers also appeared to correlate with that of the s‐SMCs, especially in corrugated structures (Figure 3B).

In contrast, incorporation of supplemented labeled fibronectin into the ECM was inhibited in high‐density and quiescent vasculature models (Figure 4B,D,F and Figure S19, Supporting Information) at the periphery of the well where the EC layer was confluent. Since the endothelium was defected in the center, the incorporation of labeled fibronectin into ECM fibers was higher. On the other hand, in c‐SMC and s‐SMC monocultures exposed to shear, the amount of labeled fibronectin normalized by SMCs was similar throughout the well (Figure S20, Supporting Information).

To verify whether there were differences in the actual synthesis of cellular fibronectin, immunostaining against total fibronectin was performed without permeabilization to avoid staining the intracellular fibronectin. The outcome was at first deceiving because it suggested that fibronectin was only synthesized in the gaps of the EC layer (Figure S21, Supporting Information). A closer look revealed though that one can find patches of fibronectin staining in regions of confluent endothelium, suggesting that the endothelial barrier could also blocking the anti‐fibronectin antibody from reaching the ECM below. Confirming this, the same patchy pattern was observed in confluent EC monocultures exposed to shear, but not on permeable ECs exposed to disturbed flow nor on s‐SMC monocultures in either static or shear conditions (Figure S22, Supporting Information). This technical issue challenges the quantification of fibronectin biosynthesis in different areas of the tissue in cocultures using immunostaining alone.

Taken together, these results illustrate the importance of a confluent endothelium in blocking the passage of large molecules that could trigger negative remodeling in the subjacent SMC layer after vascular injury, and consequently lead to IH.

### 2.8 Shear Increases EC Coverage and Island‐to‐Network Ratio in Cocultures

In the IH model, corrugated structures formed predominantly at the periphery of the well, where flow was pulsatile and laminar (Figures 1C and 3A,B). Although corrugated structures seemed to be macroscopically aligned in the direction of flow (Figure S11, Supporting Information), single s‐SMCs did not preferentially align in the direction of flow, nor did they form such prominent corrugations in shear‐exposed monocultures (Figures S12 and S23, Supporting Information).

**Figure 5:**
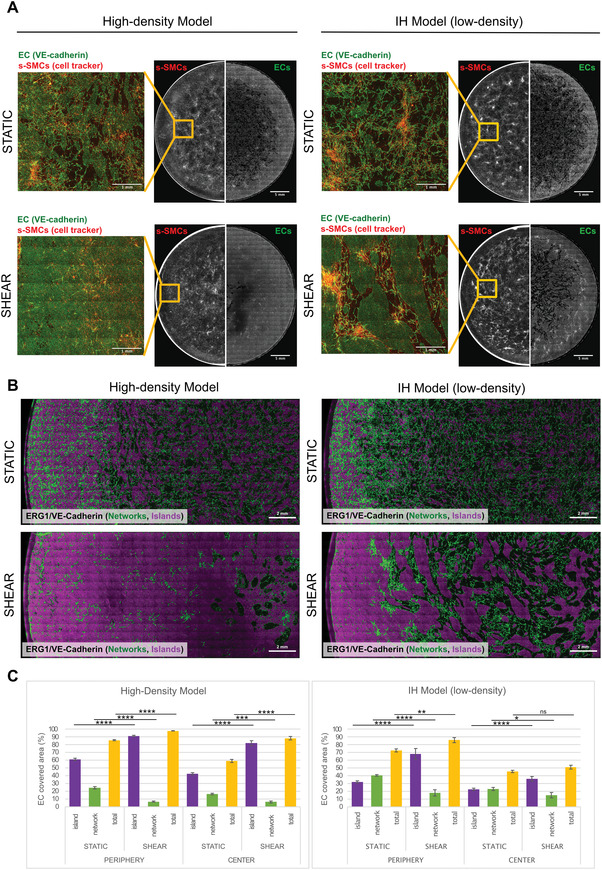
Shear increases EC coverage and island‐to‐network ratio in cocultures. ECs were seeded on top of s‐SMCs either at high density (1.5 × 10^5^ cells cm^−2^, high‐density model) or low density (8 × 10^4^ cells cm^−2^, IH model). Coculture was either exposed to shear or remained static for 3 days. A) Black and white images, acquired using widefield fluorescence microscopy, show the entire wells as large stitched mosaics. s‐SMCs stained with cell tracker (on the left half); ECs stained with VE‐cadherin antibody (on the right half). The rectangular grid patterns result from stitching. Scale bar 5 mm. Representative zoomed‐in images are shown in color from the regions marked with yellow boxes, with VE‐cadherin (green) and cell tracker (red) merged. Scale bar 1 mm. B) Output image from texture analysis distinguishes EC networks (green) from EC islands (purple). The original images acquired by widefield fluorescence microscopy show ERG1 and VE‐cadherin double immunostaining using the same secondary antibody. s‐SMCs are present, but not shown here for better visualization. Scale bar 2 mm. C) The graphs show the percentage of area covered by EC networks (green), EC islands (purple), or ECs in total (yellow). Selected regions of interest in center and periphery of images shown in (B) were analyzed, therefore showing local variance. Tukey test performed to compare static and shear conditions on each model (**** *p* < 0.001, *** *p* < 0.01, ** *p* < 0.1, *p < 0.5).

To further investigate the influence of shear in the observed corrugations, EC‐s‐SMC cocultures were grown under static conditions. Without shear, s‐SMCs in coculture with low density seeded ECs formed randomly distributed corrugated structures (**Figure** **5**). The EC layer, on the other hand, was mostly composed of abundant EC networks and various small islands. The result was similar in high‐density cocultures, but islands were slightly larger and, corrugated structures formed a smoother topography than in low‐density cocultures. These data suggest that shear is detrimental for proper regeneration and maintenance of endothelial integrity on top of s‐SMCs and that the EC network‐to‐island transition, observed in Figure 2, is delayed in the absence of shear. Moreover, this process seems to be time‐dependent, because when the same high‐density cocultures were exposed to flow at a delayed time point, i.e., after 24 hours of coculture instead of 4 hours, the EC layer was equally unable to reach confluency within the 3 days of experiment, while s‐SMC formed hill‐valley topography (Figure S24, Supporting Information). This suggests that a proliferative rate competition between EC and SMC defines whether corrugated structures will form and, that shear plays an important role in favor of ECs to close the endothelial gaps.

Interestingly, in both static and shear conditions, and at both seeding densities, a confluent endothelium was observed close to the rim of the well, suggesting that a border effect, most likely the contact guidance by the rim, stabilizes the coculture and is independent of shear (Figure 5).

### 2.9 Proof‐of‐Concept Incubation with Paclitaxel Shows Toxicity toward ECs in Cocultures under Shear

**Figure 6:**
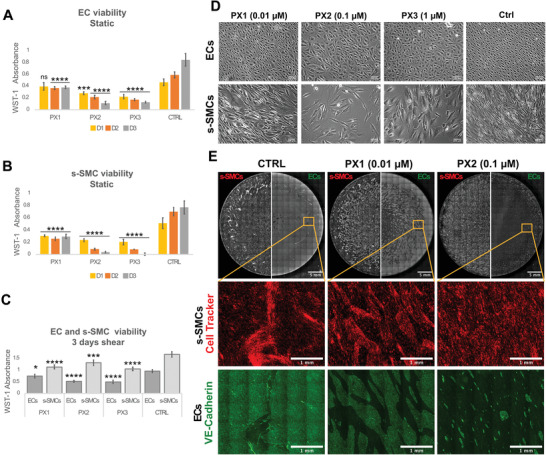
Paclitaxel reduces EC and s‐SMC viability and inhibits intimal thickening, while damaging EC monolayer integrity, which suggests a severe shortcoming when used for IH treatment. A,B) EC and s‐SMC viability measured by WST‐1 assay during 3 days in static conditions. Absorbance OD values measured at 440 nm with a reference wavelength of 650 nm. D = day; PX1 = paclitaxel 0.01 × 10^−6^ m in CCM; PX2 = Paclitaxel 0.1 × 10^−6^ m in CCM; Ctrl = Control CCM. For statistical analysis, one‐way ANOVA followed by Tukey's test was performed to compare each condition with controls (Ctrl) on the same respective day (**** p < 0.001, *** p < 0.01, ** p < 0.1, p < 0.5). C) EC and s‐SMC viability measured by WST‐1 assay after 3 days of exposure to shear on an orbital shaker. Absorbance OD values were measured at 440 nm with a reference wavelength of 650 nm. For statistical analysis, one‐way ANOVA followed by Tukey's test was performed to compare each condition with its respective control (Ctrl) (**** *p* < 0.001, *** *p* < 0.01, ** *p* < 0.1, * *p* < 0.5). D) Widefield phase‐contrast images of EC and s‐SMC monocultures grown on the orbital shaker for 3 days. Images were taken at the periphery of the well. Scale bar 200 µm. E) IH model where ECs were seeded at low density on s‐SMCs under shear for 3 days. Black and white images, acquired using widefield fluorescence microscopy, show the entire wells as large stitched mosaics. s‐SMCs stained with cell tracker (on the left half); ECs stained with VE‐cadherin antibody (on the right half). The rectangular grid patterns result from stitching. Scale bar 5 mm. Colorful images show zoomed‐in images of regions marked with yellow boxes, with VE‐cadherin (green) and cell tracker (red) merged. Scale bar 1 mm.

Since our results suggest that our IH‐on‐a‐dish model recapitulates central features of injury‐induced IH, we asked whether it could be used for in vitro drug testing and screening. Paclitaxel represents one of the most commonly used drug to suppress IH in drug‐eluting stents,^[^ ^77^ ^]^ as it stabilizes microtubules and thus inhibits cell proliferation. Therefore, we performed a proof‐of‐concept study to evaluate how different concentrations (0.01 × 10^−6^ m, 0.1 × × 10^−6^ m, and 1 × 10^−6^ m ) affect the phenomena described above in our IH model and monocultures in both shear and static conditions. In vitro, paclitaxel has been shown to have a toxic effect on s‐SMCs and ECs above 0.01 × 10^−6^ m and a cytostatic effect below.^[^ ^78^, ^79^ ^]^ In agreement with these results, on both s‐SMC and EC monocultures under static conditions, 0.01 × 10^−6^ m paclitaxel only exerted a cytostatic effect, as seen by the constant WST‐assay absorbance overtime compared to the increasing values in controls (**Figure** **6**A,B). Also under shear, 0.01 × 10^−6^ m of paclitaxel had no cytotoxic effect in both s‐SMCs and ECs, but reduced s‐SMC growth and EC alignment to shear compared to controls (Figure 6C,D). This resulted in approximately 30% and 20% difference respectively in WST absorbance after 3 days relatively to controls (Figure 6C). In cocultures after 3 days of shear exposure, 0.01 × 10^−6^ m of paclitaxel resulted in an EC monolayer with larger defects as compared to controls (Figure 6E). However, the ECs in islands still aligned in the direction of the flow and SMCs still formed corrugated structures in the regions where the endothelium was defected. This suggests that paclitaxel at a concentration of 0.01 × 10^−6^ m is not able to prevent IH but may hinder the regeneration of the endothelium.

As expected, at higher concentrations (0.1 × 10^−6^ m and 1 × 10^−6^ m), paclitaxel reduced the viability of both cell types (Figure 6A**–**C), suggesting a cytotoxic effect. Shear reduced paclitaxel's toxicity on EC monocultures, as the endothelium remained confluent, although cells lost the ability to align with the flow (Figure 6D). This is expected as paclitaxel stabilizes microtubules and microtubular integrity is essential to sense and react to shear stress.^[^ ^78^ ^]^ Furthermore, in cocultures with s‐SMCs under shear, 0.1 × 10^−6^ m had a toxic effect on the endothelium, while inhibiting the formation of corrugated structures by SMCs (Figure 6E). The toxic effect towards ECs was stronger in cocultures with a growing EC layer (Figure 6E) as compared to already confluent monocultures under shear (Figure 6D), which not only suggests that paclitaxel acts negatively on ECs during IH mainly by hindering endothelial regeneration, but also highlights the fact that EC‐SMC crosstalk can affect cellular response to drugs. The disappearance of corrugated structures with the increased concentrations of paclitaxel shows the relevance and applicability of our platform to test drug toxicity and efficacy in the prevention of IH, while controlling potential adverse effects on the EC layer.

## 3 Discussion

**Figure 7:**
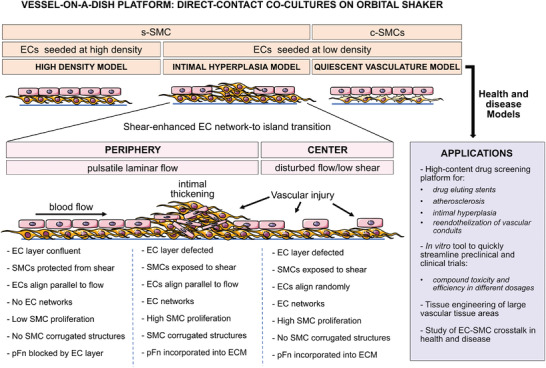
Graphical abstract summarizing the main findings of this work, the developed models, and their potential applications. Our vessel‐on‐a‐dish platform consists of direct contact cocultures placed on an orbital shaker where they got exposed to either pulsatile laminar or disturbed flow at the periphery and center of the well respectively. The high‐density coculture model consists of s‐SMCs (synthetic phenotype) topped with ECs seeded at high density, which resulted in a stable sandwich coculture. The quiescent vasculature model consists of c‐SMCs (contractile phenotype) topped with ECs seeded at low density, which grew to confluency and formed a stable coculture with the characteristics of a healthy vessel wall. The IH model consisted of s‐SMCs topped with ECs seeded at low density, which resulted in an unstable coculture. In this model, ECs formed networks on top of s‐SMCs, which overtime transitioned towards islands of confluent endothelium, which was enhanced by shear. Zoomed‐in schematics show details of the different heterogeneous regions observed in this model at the periphery and center of the well, together with the observed coculture behavior. At the periphery of the well, the EC layer consisted of regions of confluent endothelium in coexistence with EC networks. Under a confluent EC layer, SMCs were protected from shear and the formation of corrugated structures was inhibited. In regions where the EC layer was defected, SMCs were exposed to shear, increased proliferation, formed corrugated structures and incorporated plasma fibronectin (pFn) into their extracellular matrix (ECM). EC networks could also be found inside of SMC corrugated structures. At the center of the well, flow was disturbed, causing ECs to align randomly and larger defects on EC layer. EC networks were observed, and SMC proliferation was high, although no pronounced corrugated structures were formed by SMCs. Since the EC layer was defected, available pFn was incorporated into the ECM by SMCs. The utilization of such a platform, together with the three developed models, can be explored as a high‐content drug screening platform. Potential applications include the testing of drugs for the treatment of atherosclerosis, IH inhibition in vascular grafts and stents, reendothelialization of vascular conduits, and others. Such an in vitro tool allows to quickly streamline protocols for preclinical and clinical trials, by providing means to test efficacy and toxicity at different dosages. It could also be a helpful tissue engineering platform to develop more complex protocols, by including levels of complexity such as a basement membrane, or to test new materials for tissue engineering applications. Furthermore, such model creates not only the possibility to address basic questions related to SMC‐EC crosstalk at the cellular and tissue level in health and disease, but also to pinpoint the mechanisms behind IH onset.

Here we present a vessel‐on‐a‐dish platform consisting of direct contact cocultures of ECs and SMCs in 6‐well plates and exposed to shear on an orbital shaker. The different models (“quiescent,” “intimal hyperplasia,” and “high density”) resulted from different SMC phenotypes (synthetic or contractile) and different EC seeding densities, as summarized in **Figure** **7**.

In the model of quiescent vasculature, ECs were seeded on top of c‐SMCs mimicking the architecture of a healthy vessel wall (Figure 1A,B).^[^ ^80^ ^]^ Similar direct contact cocultures with c‐SMCs have been created before,^[^ ^55^, ^56^, ^57^, ^59^, ^66^, ^81^ ^]^ but our model adds novelty since it is the first EC‐SMC coculture exposed to shear for days on an orbital shaker and with large tissue areas analyzed. ^[^ ^41^ ^]^

Our novel IH model (Figure 1A,C), replicates a region of injury denuded from ECs and basement membrane, with activated SMCs exposed to flow, but no immune cells. Vascular injury and reendothelization are replicated through incomplete EC spreading, since a scratch assay would damage the SMC layer underneath. Thus, EC seeding on top of s‐SMCs mimics the in vivo arrival of ECs to an injury site through migration from neighboring regions or endothelial progenitor cells from the blood stream.^[^ ^82^ ^]^ While the ECs had grown into confluency on c‐SMC (Figure 1B and Figure S3A, Supporting Information), the EC layer was still defected on s‐SMCs in the IH model after 3 days of coculture under shear (Figures 1C, 2, and 3A,B). This highlights that the EC layer regenerates better in contact with c‐SMCs compared to s‐SMCs, as supported by our quantifications of proliferating ECs (Figure S3B, Supporting Information) and as suggested before. ^[^ ^59^, ^66^, ^67^ ^]^ Possible reasons could be the phenotype‐specific secretion of different paracrine factors or direct‐contact signaling, or the fact that fibronectin ECM is more abundant in c‐SMCs induced with TGFßM (Figure S2E,F, Supporting Information). Cell adhesion, migration and proliferation are enhanced on fibronectin, and specifically, ECs have been shown to spread slower on SMCs than attached to fibronectin surfaces.^[^ ^83^, ^84^ ^]^

The whole tissue reconstructions as large tile mosaics that were shown throughout this study, have allowed to appreciate the true complexity and mesoscale heterogeneity of the resulting cocultures (e.g., Figures 1C, 2A and 3A and Figure S11, Supporting Information). Specially in our novel IH model, a series of interesting phenomena and regional differences (summarized in Figure 7) could have been missed if only representative single images had been taken. ECs organized into two coexisting assemblies: 2D islands of confluent ECs with the typical cobblestone morphology and EC networks consisting of elongated ECs (Figure 2). Such EC networks have been well described in several gels and on top of various cell types,^[^ ^69^, ^70^, ^71^, ^72^, ^73^ ^]^ and their formation depends on various biochemical and mechanical factors such as substrate stiffness, interactions with neighboring cells or ECM and cell–cell signaling.^[^ ^85^ ^]^ Interestingly, the area covered with EC networks was reduced over time while the 2D EC islands grew in size, in a cell density‐dependent manner (Figure 2 and Figure S10, Supporting Information). This morphogenetic transition has not been described before either in vivo or in vitro and, seems to be promoted by shear, since in static conditions the islands were smaller and EC networks more abundant compared to flow conditions (Figure 5). These results underline the importance of shear in vascular reendothelialization, in line with previous reports.^[^ ^8^, ^33^ ^]^ Moreover, despite the observed network‐to‐island transition, the total area covered with ECs changed minimally over the 3 days of coculture (Figure 2D and Figure S3B, Supporting Information). This might suggest that contrary to the island growth typically seen on bare culture dishes (Figure S4, Supporting Information), on s‐SMCs elongated ECs may migrate in the form of networks to find neighboring ECs, and only after those contacts are created, they may adapt the well‐known cobblestone morphology to form a 2D monolayer. Whether this provides insights how an injury impacted endothelium might regenerate in a 3D environment in vivo could be followed up in future studies.

Remarkably, the s‐SMC layer thickened locally in the IH model (Figures 1C and 3A–C), forming what we here referred to as “corrugated structures” (Figure 3C and Figure S11 and Videos S3 and S4, Supporting Information). These resulted from an increase in s‐SMC proliferation in regions of endothelial defects (“gaps”) (Figure 3D), most likely combined with cell migration.^[^ ^17^ ^]^ Due to a possible higher concentration of locally produced attractants by ECs,^[^ ^28^, ^36^ ^]^ the s‐SMCs might even migrate over EC networks, entrapping them inside corrugated structures (Figure 3C‐b,E; Videos S2A and S3B, Supporting Information). On the contrary, SMC proliferation and formation of corrugated structures was inhibited below EC islands, in both IH and high‐density models (Figures 1B, 3, and 4A–D,G, and Figures S9, S15, and S10D, Supporting Information). Further, in the high‐density model, expression of the SMC contractile marker *α*SMA ^[^ ^12^, ^58^ ^]^ increased below the confluent EC layer (Figure 4E and Figure S5, Supporting Information), which might indicate an important role of the regenerated endothelium on reverting SMC phenotype after injury. SMC proliferation may be inhibited due to a combination of EC‐SMC direct contact signaling and the diffusion barrier created by tight EC‐EC junctions preventing large molecules from reaching the SMCs underneath, the latter illustrated in Figures 3A and 4B and Figures S21 and S22 (Supporting Information). Inhibition of SMC proliferation by confluent ECs has also been reported in both static and shear‐exposed coculture systems on opposite sides of a membrane, with SMC proliferation increased in partly denuded EC regions,^[^ ^33^, ^86^ ^]^ which supports our work (Figures 3D and 4G). This dual effect may result from the fact that quiescent and proliferative ECs, in the respective confluent versus defected regions, secrete different factors and activate different signaling pathways that consequently differently regulate the subjacent SMCs.^[^ ^28^, ^86^ ^]^ Taken together, our results suggest that corrugated structures result from combined inhibitory and stimulatory direct‐contact and paracrine signaling between ECs and SMCs under shear. Our model recapitulates well‐known early steps of IH onset, such as the increase in SMC proliferation and the consequent intimal thickening after vascular injury,^[^ ^17^ ^]^ and highlights the importance of EC‐SMC communication in IH, even in the absence of coagulation factors and the immune system.

Another important take home message is that even in the presence of the “destabilizing” s‐SMCs, a stable “sandwich” architecture can be achieved by seeding ECs at high density and exposing the cocultures to shear (Figures 4 and 5). This is particularly relevant for the field of vascular tissue engineering that is aiming at the in vitro creation of large tissue areas for endothelialization of vascular grafts of cardiovascular devices.^[^ ^87^ ^]^ Thus, the high‐density model confirmed the importance of EC seeding density in the stability of EC‐SMC direct contact cocultures, as suggested before.^[^ ^59^ ^]^ These results support the premise that a quick reendothelialization may inhibit IH (corrugated structures), since a confluent endothelium alone (EC islands) can suppress SMC proliferation (Figures 3D and 4A–D,F,G), which was further supported by various in vivo studies.^[^ ^88^, ^89^, ^90^, ^91^, ^92^, ^93^, ^94^, ^95^ ^]^

In vivo , the endothelium protects the SMCs from exposure to synthetic phenotype‐inducing blood components, such as thrombin, platelet‐derived growth factors, and leukocyte‐derived cytokines.^[^ ^96^ ^]^ As illustrated in our quiescent vasculature and high‐density models, the tight EC–EC junctions also block SMCs access to added fluorescently labeled plasma fibronectin (Figure 4B,F and Figure S19, Supporting Information). On the contrary, in our IH model, major fibronectin assembly was observed in the defected regions of the EC layer (Figure 3A,B,F). As plasma fibronectin lacks the alternatively spliced EIIIA and EIIIB domains of cellular fibronectin,^[^ ^97^ ^]^ these isoforms play different roles during vascular injury and regeneration.^[^ ^98^ ^]^ In fact, the concentration of soluble plasma fibronectin is increased in patients with cardiovascular disease.^[^ ^99^, ^100^ ^]^ As illustrated in our models, SMCs have access to plasma fibronectin only after endothelial denudation (Figures 3A,B,F and 4B), which gets coassembled with cellular fibronectin into ECM fibrils and serve as a fiber tension dependent template for other proteins, such as collagen.^[^ ^76^ ^]^ ECM deposition characterizes the 3^rd^ phase of IH development,^[^ ^17^ ^]^ thus such early ECM assembly events could determine whether this process ends in regeneration towards a healthy vessel wall or pathological IH.

Our data not only provide physiologically significant insights but should provoke a rethinking of strategies to suppress IH. Although current clinically available preventive therapies primarily rely on antiproliferative strategies,^[^ ^19^ ^]^ here we show that concentrations of paclitaxel that are necessary to inhibit IH may worsen the local denudation of the endothelium, even in the presence of physiological shear and close proximity with SMCs (Figure 6E). In fact, other in vitro studies have shown that paclitaxel reduces cell proliferation, migration, capillary network formation in a dose‐dependent manner and increases apoptosis of ECs.^[^ ^101^ ^]^ A link between denudation and thrombosis has been established ^[^ ^102^ ^]^ and clinical evidence shows that drug‐eluting stents may potentiate late thrombosis due to delayed reendothelialization.^[^ ^103^ ^]^ Even more alarming, discontinuation of antiplatelet therapy in patients who have received paclitaxel and sirolimus‐coated stents is associated with an increased risk of late in‐stent thrombosis. ^[^ ^22^ ^]^ Following several randomized clinical trials^[^ ^20^ ^]^ that showed an association between paclitaxel‐coated balloons and stents with an increased risk of late morbidity compared to their uncoated counterparts, the Food and Drug Administration (FDA) released a warning of caution when utilizing such devices.^[^ ^21^ ^]^ Thus, strategies that enhance EC regeneration, rather than block SMC and EC proliferation, may be more promising in the prevention of IH. This hypothesis has also been supported by various IH‐related in vivo studies and new approaches have been suggested.^[^ ^88^, ^89^, ^90^, ^91^, ^92^, ^93^, ^94^, ^95^ ^]^

As exemplified by our proof‐of‐principle experiment with paclitaxel (Figure 6), it should be quite straight forward to use our model to test drug candidates for IH treatment or prevention. A promising drug candidate will result in a confluent EC layer while hindering the appearance of SMC corrugated structures. Thus, only two parameters are relevant 1) confluency of EC layer, as an indicative for whether the drug may injury the endothelium or delay/accelerate reendothelization, and 2) presence or absence of corrugated structures, to confirm the drug's ability to prevent IH. Such complex cocultures of human cells present an advantage as opposed to 2D monocultures, since they allow to study the effect of each drug candidate on the fine balance between EC regeneration and SMC proliferation (Figure 6D,E). Although concentrations cannot be directly compared with in vivo scenarios, such an in vitro tool provides means to test efficacy and toxicity at different dosages and thereby improving the pre‐selection of promising drug targets for the preclinical and clinical phases.

Although our setup on the orbital shaker cannot mimic arterial pressure nor blood viscosity, it is an easy‐to‐implement and scalable method to expose cells to pulsatile flow, with low associated cost and no need of any complicated equipment or engineered set‐ups. Our platform allows fine‐tuning of the pulse frequency and the magnitude of shear stress, by varying the well diameter, medium volume, medium viscosity, and rotation speed, which might be relevant for future investigations. By virtue of the simplicity of the dish‐based culture system, the model still has a considerable potential to increase levels of complexity to address further questions relevant in vivo, e.g., by adding or allowing basement proteins to be secreted and assembled, by adding inflammatory cytokines, or performing triple coculture with leukocytes or macrophages.

Our IH model, based on common laboratory‐level instruments and operations, goes far beyond the currently available in vitro IH models by exploring EC‐SMC interactions while exposed to shear and by analyzing large tissue areas. While the use of animal models is time consuming, expensive, and only enable the study of complex tissues at defined endpoints, our model allows continuous monitoring of tissue rearrangement over time due to cell–cell and cell–matrix interactions. It offers a relatively quick screening method and a fast readout for drug testing since corrugated structures can be seen by eye after 2–3 days (Figure S11, Supporting Information). The screening potential could be maximized by using the multistaining platform that we have developed precisely for this set up, allowing to perform several simultaneous stainings on each well, as we did on Figure S5 (Supporting Information).^[^ ^104^ ^]^ Further, this platform could also be used as a tool to design protocols for mimicking in situ reendothelialization of bioengineered vascular conduits or heart valves,^[^ ^105^ ^]^ endothelialization of implant surfaces and/or test the interaction of different biomaterials with both health and disease models.

## 4 Conclusion

Our platform and the developed health and disease vascular models have therefore a high clinical and translational relevance, offering a new tool to better understand and prevent injury‐related IH and the resulting restenosis and in‐stent thrombosis. Further, it is of significant value for fundamental biologists and bioengineers, as well as for the pharma and the medical device industry.

## 5 Experimental Section

### Cell Culture

Human umbilical endothelial cells (HUVECs) were kindly isolated at University Zurich by Dr. Sarah Motta and provided by Prof. Hoerstrup (ethical approval from the Kantonale Ethics Commission Kanton Zürich Nr. 2016‐00208). Cells were expanded up to passage 6 in EGM‐2 medium supplemented with EGM‐2 BulletKit (CC‐3162, Lonza). Human Aortic Smooth Muscle Cells (HASMC) were purchased from Lonza and expanded up to passage 7 in SmGM medium supplemented with Smooth Muscle Cell Growth Medium 2 Kit (CC‐3182 Lonza or C‐22162 Promocell).

### Induction of Synthetic and Contractile Phenotype

At passage 8, HASMCs were seeded on 0.2% gelatin‐coated glass or plastic‐bottom six‐well plates with a cell density of 4 × 10^4^ cells cm^−2^, using SmGM. After 24 hours, the medium was exchanged to the respective medium to induce either the synthetic (s‐SMC) or contractile (c‐SMC) phenotype during 3–4 days in static conditions. Unless stated otherwise, s‐SMC cells were grown in SmGM, whereas c‐SMC cells were cultured In TGFßM (see Table 1) prior to seeding the ECs. Medium was exchanged every 24 hours.

### Coculture Experiments

Immediately before starting coculture, the confluent cell layer of either c‐SMCs or s‐SMCs was stained with 10 × 10^−6^ m of CellTracker Green CMFDA Dye (Thermofisher C7025) diluted in MCDB131 medium (10372019, Invitrogen) supplemented with 1× insulin‐transferrin‐selenium (41400045, Gibco) and 1× Glutamax (35050061, Gibco). Since washing with a saline solution such as PBS can cause tissue detachment, after a 30–40 min incubation with cell tracker at 37 °C, SMCs were quickly washed with Hepes Buffered Saline (NaCl, KCl, Na_2_HPO_4_·2H_2_O, HEPES), followed by 2 long washes in either SmGM or CCM. HUVECs were seeded on top of HASMCs with a cell density of either 8 × 10^4^ (low density seeding) or 1.5 × 10^5^ cells cm^−2^ (high density seeding), using CCM (see Table 1). After 4 hours to allow EC adhesion, medium was exchanged with 2 mL of fresh CCM and placed on an orbital shaker (VWR Advanced 3500 Orbital Shaker, radium 9.5 mm) at 135 rpm. When indicated, other coculture media were used: m200, EGM‐2 or CCM without the addition of EGF and FGFb (CCM‐EGF‐FGFb) (see Table 1). Every 24 hours during the 3 day experiment, 50% of medium volume was collected and stored and fresh medium was added, always with extra 100 µL to compensate for evaporation. Static controls were cultured on the same incubator and went through the same process as flow‐induced cultures.

### Fibronectin Experiments

Plasma fibronectin was isolated in the laboratory, according to Früh et al.^[^ ^106^ ^]^ Fibronectin was labeled using Alexa Fluor 647 NHS Ester (succinimidyl ester) (Invitrogen A20106), resulting in a degree of labeling of 10 (10 molecules of dye per molecule of fibronectin). During coculture, labeled fibronectin was added every 24 hours in the medium at a concentration of 5 mg mL^−1^. On day 2 and 3 of coculture, only 50% of culture medium was exchanged by fresh medium containing labeled fibronectin.

### Paclitaxel Experiments

Paclitaxel (Sigma, T7402‐1MG) was diluted in DMSO to a stock concentration of 10 × 10^−3^ m. For static studies, ECs and s‐SMCs were seeded at 6 × 10^3^ cells cm^−2^ density on 0.2% gelatin‐coated TPP plastic bottom 96 well plates using CCM. During 3 days, cells were cultured in static conditions and fresh medium was supplemented daily with fresh paclitaxel (0.01 × 10^−6^ m, 0.1 × 10^−6^ m, and 1 × 10^−6^ m). Three independent plates were prepared to measure metabolic activity with WST‐1 assay (Roche, Cat. No. 11644807001) at 24 hours, 48 hours, and 72 hours. WST‐1 was diluted 1:10 into each well, incubated for 1.5 hours and absorbance was read at 440 nm with a reference wavelength of 650 nm. For shear‐exposed cells, ECs and s‐SMCs were seeded on glass‐bottom 6‐well plates at 8 × 10^4^ cells cm^−2^ and 4 × 10^4^ cells cm^−2^ seeding density, respectively. After 24 hours, plates were placed on the orbital shaker and exposed to flow for 3 days on the orbital shaker in CCM. EC layer was confluent when placed on the shaker. Every day, fresh medium was exchanged containing paclitaxel at the above‐mentioned concentrations. Additionally, phase‐contrast images were acquired in the peripheral region of the well every 24 hours. After 72 hours, cells were fixed with 4% paraformaldehyde.

For coculture experiments, ECs were seeded on top of cell tracker labeled s‐SMCs at low density (8 × 10^4^ cells cm^−2^), as described in Figure 1A. After 4 hours, cocultures were placed on the orbital shaker for 3 days. Every day, 50% of the medium was exchanged by fresh CCM containing paclitaxel at 0.01 × 10^−6^ m and 0.1 × 10^−6^ m. After 3 days, cells were fixed and immunostained against VE‐cadherin, according to the protocol described in Supporting Information and methods. Further protocols and antibody details are provided in Supporting Information.

## Conflict of Interest

The authors declare no conflict of interest.

## Supporting information

Supporting Information

Supplemental Video S1

Supplemental Video 2A

Supplemental Video 2B

Supplemental Video 3A

Supplemental Video 3B

Supplemental Video 4

Supplemental Video 5A

Supplemental Video 5B

Supplemental Video 6

## Data Availability

The data that support the findings of this study are available from the corresponding author upon reasonable request.
