# Supplementary material for: Endothelial‐Smooth Muscle Cell Interactions in a Shear‐Exposed Intimal Hyperplasia on‐a‐Dish Model to Evaluate Therapeutic Strategies
Source: Adv Sci (Weinh). 2022 Aug 15;9(28):2202317. doi: 10.1002/advs.202202317 (PMC9534971; doi:10.1002/advs.202202317)
Supplement: Supplementary file 1 — Supporting Information [file ADVS-9-2202317-s007.pdf]

ADVANCED SCIENCE
Open Access

Supporting Information

for *Adv. Sci.*, DOI 10.1002/advs.202202317

Endothelial-Smooth Muscle Cell Interactions in a Shear-Exposed Intimal Hyperplasia on-a-Dish Model to Evaluate Therapeutic Strategies

*Andreia Fernandes, Arnaud Miéville, Franziska Grob, Tadahiro Yamashita, Julia Mehl, Vahid Hosseini, Maximilian Y. Emmert, Volkmar Falk and Viola Vogel**

Supporting Information and Methods

Endothelial-Smooth Muscle Cell Interactions in a Shear-Exposed Intimal Hyperplasia on-a-Dish Model to Evaluate Therapeutic Strategies

*Andreia Fernandes, Arnaud Miéville, Franziska Grob, Tadahiro Yamashita, Julia Mehl, Vahid Hosseini, Maximilian Y. Emmert, Volkmar Falk, Viola Vogel**

Table of Contents

1. SUPPLEMENTARY DATA -- Page 2

2. SUPPLEMENTARY METHODS --- Page 30

3. SUPPLEMENTARY VIDEOS -- Page 36

1. SUPPLEMENTARY DATA

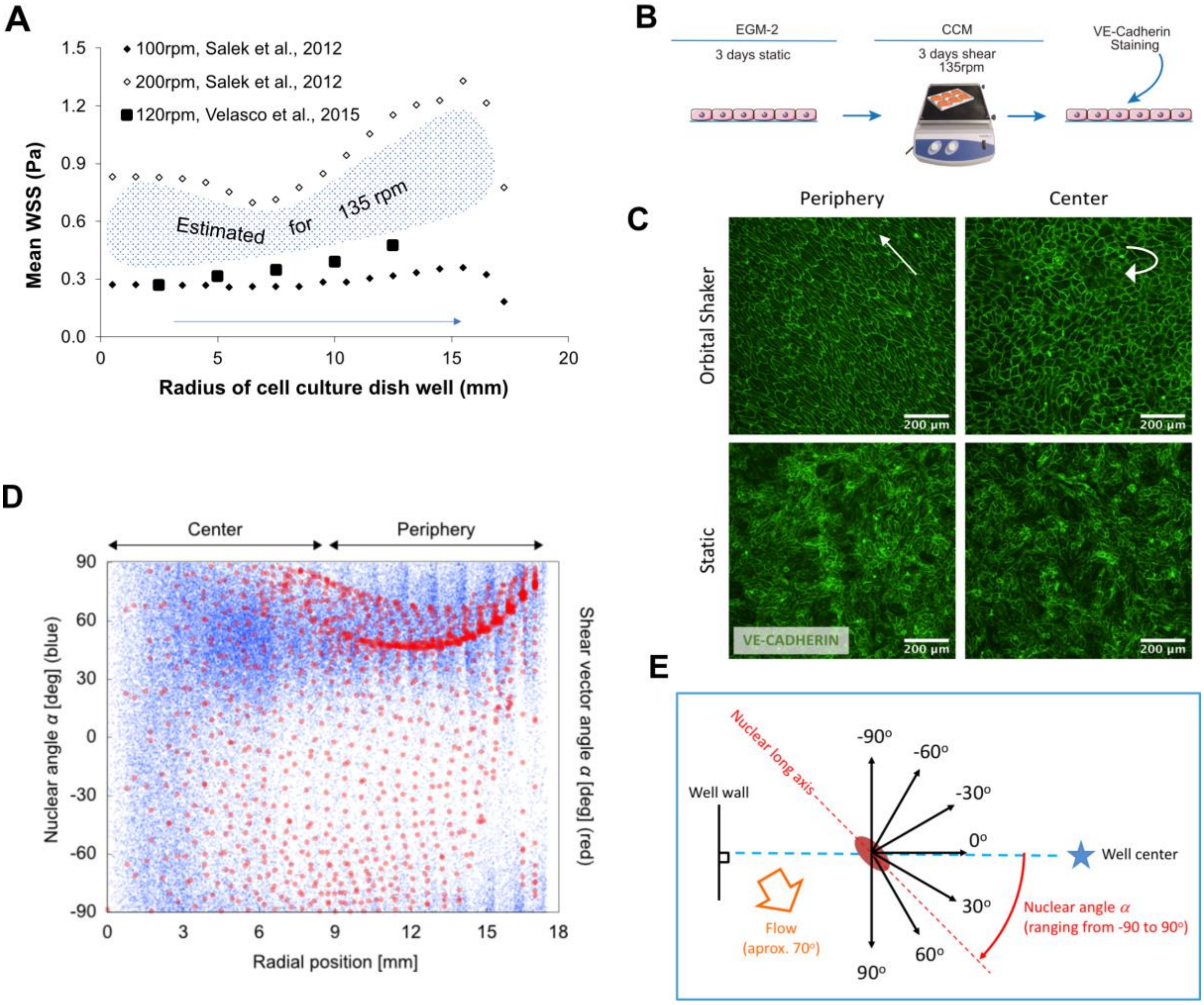

Figure S1 – Endothelial cells align with the flow after exposure to shear on the orbital shaker. (A) Estimated mean wall shear stress (WSS), in Pascal (Pa), from the center to the periphery of the well [43] for 100 rpm and 200 rpm and for 120 rpm [44]. The shaded area represents the estimated range of mean WSS at the conditions used in our experiments, i.e. 135 rpm. All conditions assume that fluid volume is 2 ml per well. **(B)** Schematics describing the protocol to create EC monocultures exposed to shear as in (C). Human Primary Umbilical Vein ECs (HUVECs) were grown for two days in Endothelial Growth Medium (EGM-2, Table 1) in static conditions until reaching confluency. Cells were transferred to the orbital shaker and grown for 3 days in 2 ml of coculture medium (CCM, Table 1) and 135 rpm rotation speed. For static cocultures, the protocol was identical except that cells were not placed on the shaker. **(C)**

HUVEC monoculture after 3 days in CCM either on the orbital shaker or in static conditions, according to protocol described in (B). Images were taken in both center and periphery of the well and show cell-cell junctions through VE-cadherin immunostaining (green). As a reference, the well wall is on the left side. Straight white arrow indicates the direction of the flow, while curved arrow represents disturbed flow. Scale bar 200µm. **(D)** Graph showing the distribution of EC nuclei orientation in blue (angle α, degrees°) from center to periphery of the well. Orientation of nuclei stained with DAPI were analyzed using a Matlab script. Angle α is defined as indicated in (E). Red dots represent the angle α of shear vectors that were previously published[43] and kindly provided by the author. Blue dots that co-colocalize with red dots represent cells that are aligned with the flow. The vertical white patterns in the blue dots are an artifact derived from image stitching. **(E)** Schematics explaining definition of angle α used in graph (D). Angle α is defined as in Salek et. al. [43] for better comparison with computer simulations.

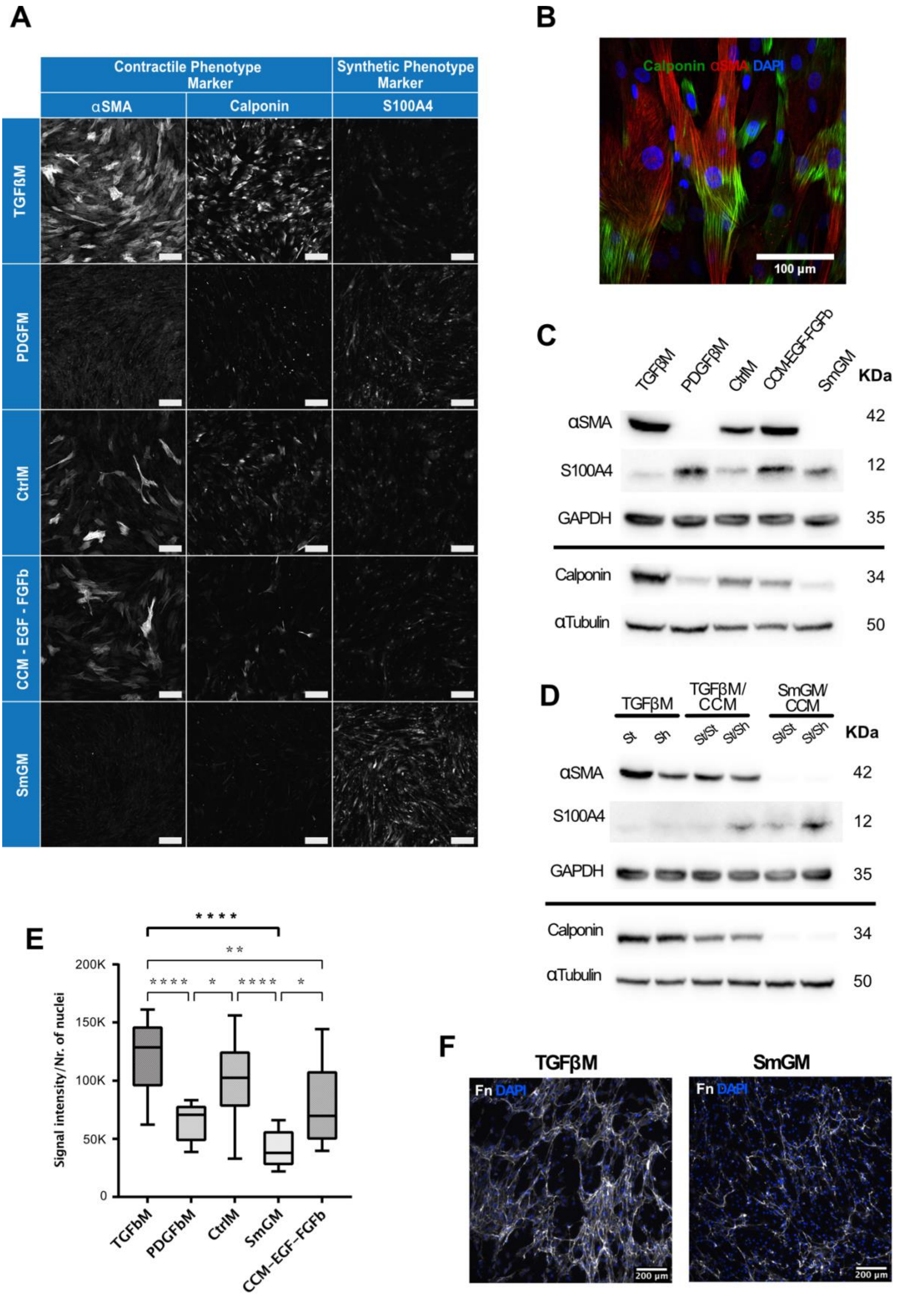
A
Contractile Phenotype Marker
Synthetic Phenotype Marker
αSMA
Calponin
S100A4
TGFßM
PDGFM
CtrlM
CCM - EGF - FGFb
SmGM
B
Calponin αSMA DAPI
100 µm
C
TGFβM
PDGFβM
CtrlM
CCM-EGF-FGFb
SmGM
KDa
αSMA
42
S100A4
12
GAPDH
35
Calponin
34
αTubulin
50
D
TGFβM
TGFβM/ CCM
SmGM/ CCM
St
Sh
St/St
St/Sh
St/St
St/Sh
KDa
αSMA
42
S100A4
12
GAPDH
35
Calponin
34
αTubulin
50
E
Signal intensity/Nr. of nuclei
200K
150K
100K
50K
0
****
**
****
*
****
*
TGFbM
PDGFbM
CtrlM
SmGM
CCM-EGF-FGFb
F
TGFβM
SmGM
Fn DAPI
200 µm

Figure S2 – Characterization of the effect of different culture media on the SMC phenotype. Human aortic SMC monocultures were cultured under static conditions, for 3 days with different culture media (**Table 1**) for manipulation of phenotype. Contractile Phenotype Inducing Medium (TGFβM); Synthetic Phenotype Inducing Medium (PDGFM); Control Medium (CtrlM); Coculture medium without growth factors (CCM-EGF-FGFb); Smooth Muscle Growth Medium (SmGM). **(A)** The immunofluorescence confocal images show the expression of contractile phenotype markers αSMA and calponin, as well as of the synthetic marker S100A4. Scale Bar 200µm. **(B)** Higher resolution confocal image of SMCs treated with TGFβM for 3 days in static conditions and expressing contractile phenotype markers αSMA (red) and Calponin (green), as well as DAPI (blue). NOTE: Similar images have been shown in Beamish et al. [10] **(C)** Human aortic SMC monocultures were cultured under static, for 3 days with different culture media (**Table 1**) for manipulation of phenotype, as in (A). The western blot shows protein levels of αSMA (42KDa) and S100A4 (12 KDa), using GAPDH (35 KDa) as a loading control in a 12% gel. Calponin (34 KDa) was blotted using alpha-Tubulin (50 KDa) as a loading control in a 10% gel. **(D)** Since EGF and FGFb in CCM and shear are known to induce the synthetic phenotype, we tested whether TGFβ-induced contractile SMCs shifted back to synthetic after 3 days on the orbital shaker in CCM. This experiment served as a control for the stability of the SMC phenotype, since cocultures with ECs were performed under the same conditions. Thus, SMC monocultures were cultured in TGFβM for 3 days in either static conditions (St) or exposed to shear (Sh). Medium was then exchanged to coculture medium (CCM) and cells were cultured for additional 3 days in either shear (St/Sh) or static conditions (St/St). As a control, SMC monocultures were grown in SmGM medium and then transferred to CCM for additional 3 days in either static (St/St) or shear (St/Sh) conditions. Protein levels of αSMA (42KDa) and S100A4 (12 KDa) were blotted, using GAPDH (35 KDa) as a loading control in a 12% gel. Calponin (34 KDa) was blotted using alpha-Tubulin (50 KDa) as a loading control in a 10% gel. **(E)** Human aortic SMC monocultures were cultured under

static conditions, for 3 days with different culture media, as in (A). Graph shows quantification of extracellular fibronectin, through immunostaining (intensity of signal/number of nuclei). **(F)** As example of images used for quantification in (E), the immunofluorescence confocal images show fibronectin immunostaining (gray) and DAPI staining (blue) in SMC monocultures after 3 days in either TGFβM or SmGM under static conditions. Scale Bar 200µm.

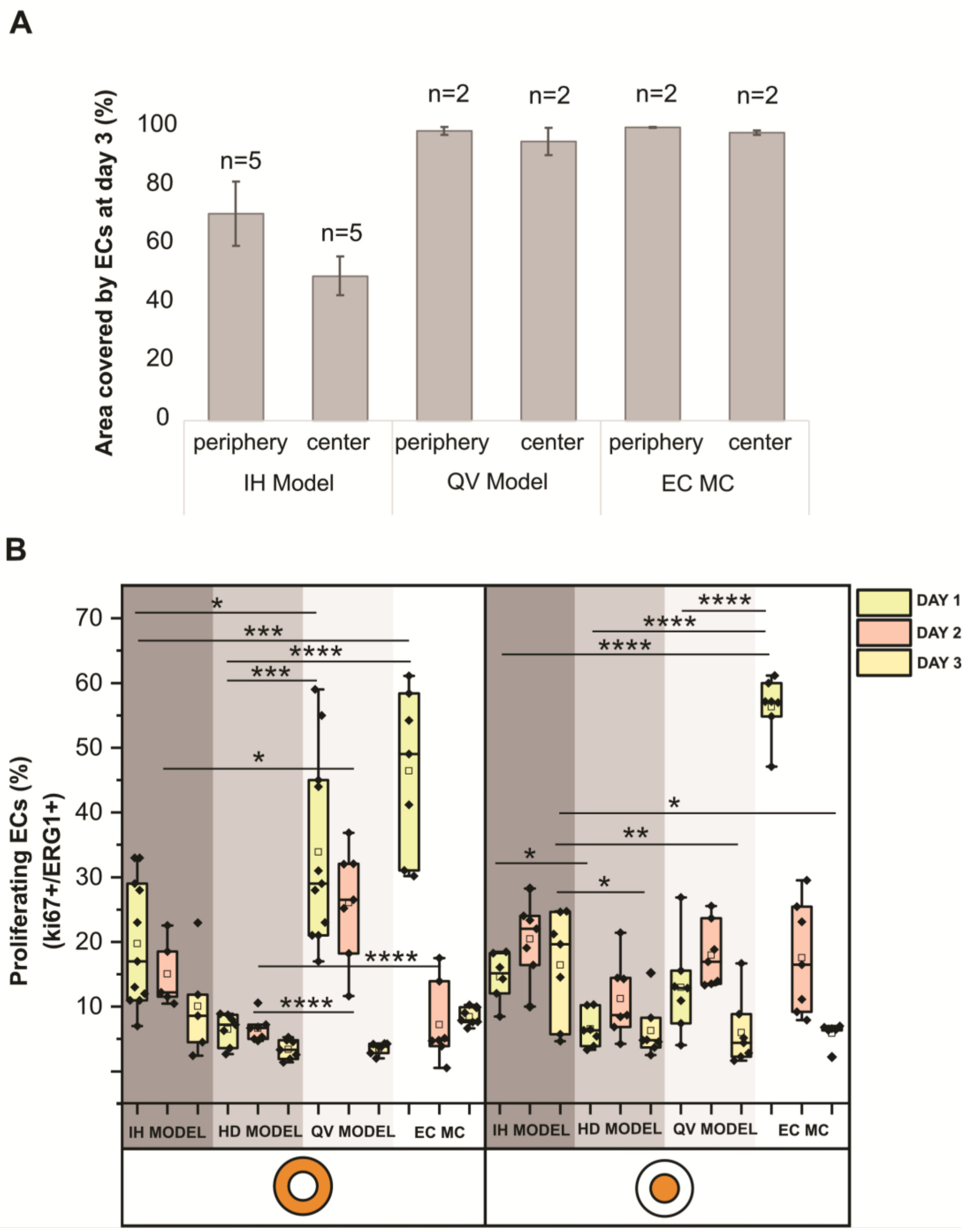

Figure S3 – Proliferation of ECs in the different vessel-on-a-dish models and the resulting percentage of covered area. (A) Area covered by ECs in the IH model, Quiescent Vasculature (QV) model and EC monoculture (EC MC) after 3 days on the orbital shaker in coculture medium (CCM, **Table 1**), as described in **Figure 1A**. Total area covered with ECs was calculated using the large mosaic images showing VE-cadherin staining in the whole well or only half of the well (images not shown). VE-cadherin fluorescence signal was blurred and binarized for area calculation using Fiji software. Percentage of tissue covered with ECs was calculated based on the total area of the tissue, using Excel. Periphery and center regions of the well were compared. Two samples (n=2) or five samples (n=5) per condition were analyzed. **(B)** Percentage of proliferating ECs in the IH model, Quiescent Vasculature (QV) model and EC monoculture (EC MC) at day 1, 2 and 3 of coculture on the orbital shaker and comparison between center and periphery of the well. For all conditions CCM medium was used (see **Table 1**). To distinguish from SMCs, all EC nuclei were stained with ERG1 antibody. Proliferative cells were immunostained with anti-ki-67 antibody, and all nuclei were stained with DAPI. Nuclei were counted using a script in Fiji/ImageJ. For statistical analysis, one-way ANOVA followed by Tukey's test was performed to compare between different models within the same time point and region of the well. **** $p<0.001$, *** $p<0.01$, ** $p<0.1$, *$p<0.5$).

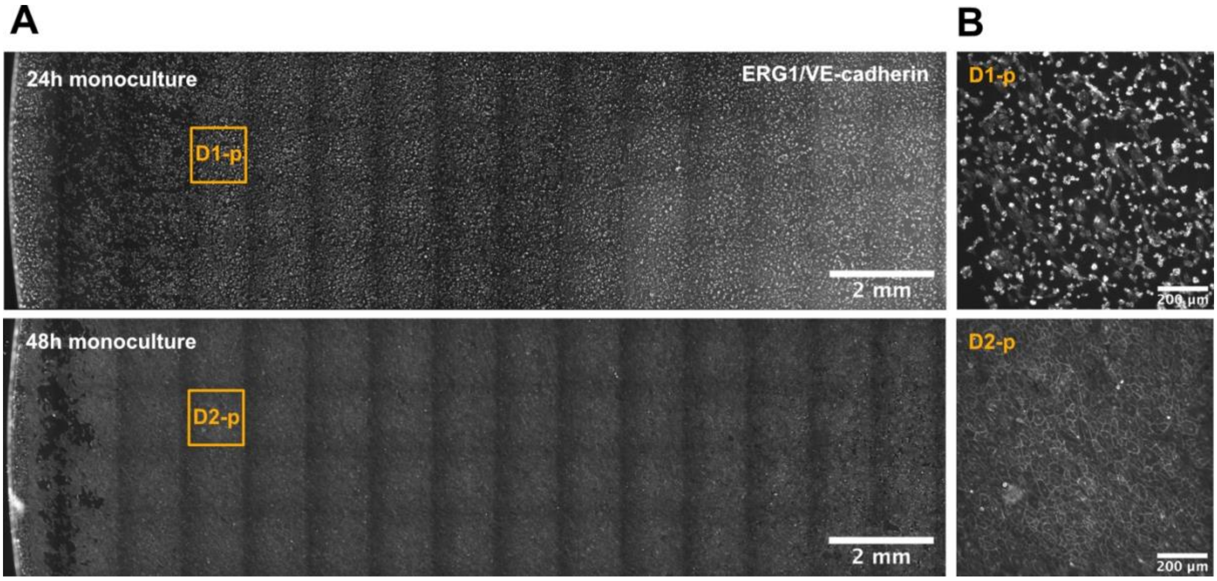

Figure S4 – Growth of ECs in monoculture exposed to shear. ECs were seeded at 8x10^4 cells/cm^2 and exposed to shear for 3 days, such as in IH and Quiescent Vasculature models. **(A)** Widefield fluorescence images show a fraction of the tissue, from border to the center of the well, after 24h and 48h on the orbital shaker. Each image belongs to an independent sample. Double Immunostaining for EC nuclei (ERG1) and cell-cell junctions (VE-cadherin) using the same secondary antibody. Scale bar 2mm. **(B)** Zoomed-in images of regions marked with yellow box in (A), showing double immunostaining for ERG1 and VE-cadherin. Day 1 - periphery (D1-p); Day 2 - periphery (D2-p). Scale bar 200µm.

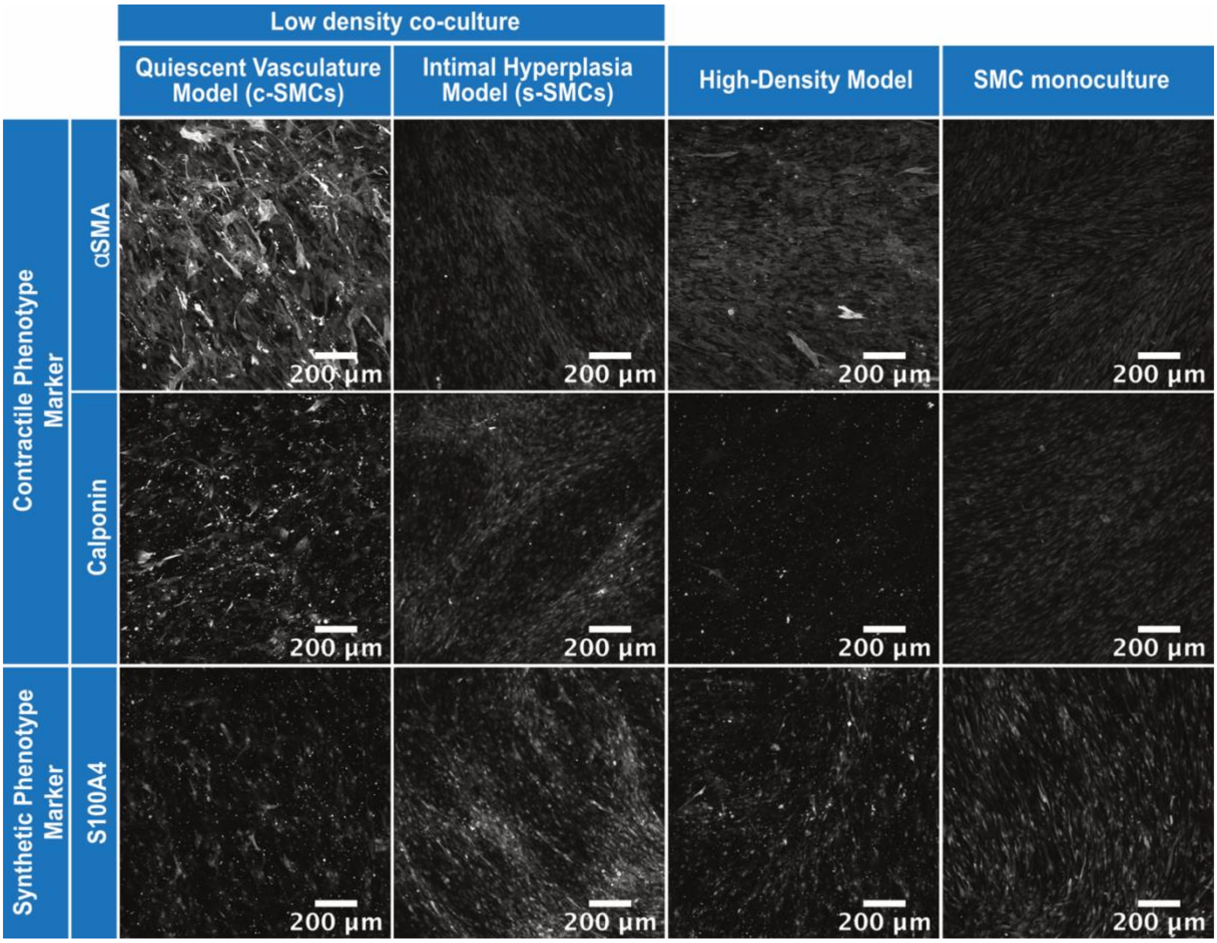

Figure S5 – SMC phenotype markers in the different models after 3 days on an orbital shaker. To investigate the phenotype of SMCs using different co-culturing conditions, ECs were seeded on top of SMCs and cultured for 3 days on the orbital shaker in CCM (**Table 1**) as described in **Figure 1A** and **4.** Quiescent vasculature model: ECs seeded at low density on top of c-SMCs. Intimal hyperplasia model: ECs seeded at low density on top of s-SMCs. High-density coculture: ECs seeded at high density on top of s-SMCs. SMC monocultures were also cultured in CCM for 3 days on the orbital shaker. The immunofluorescent images, acquired with an Olympus FV1000 laser scanning confocal microscope, show the expression of contractile phenotype markers, i.e. αSMA and calponin and, of synthetic marker S100A4. The three different stainings were performed on the same sample but at different sites. Compartmentalization of the well for these 3 independent stainings was possible with our multi-

staining platform, described in *Fernandes et al. 2022.* [104] Images were acquired at the periphery of the well. Scale bar 200µm.

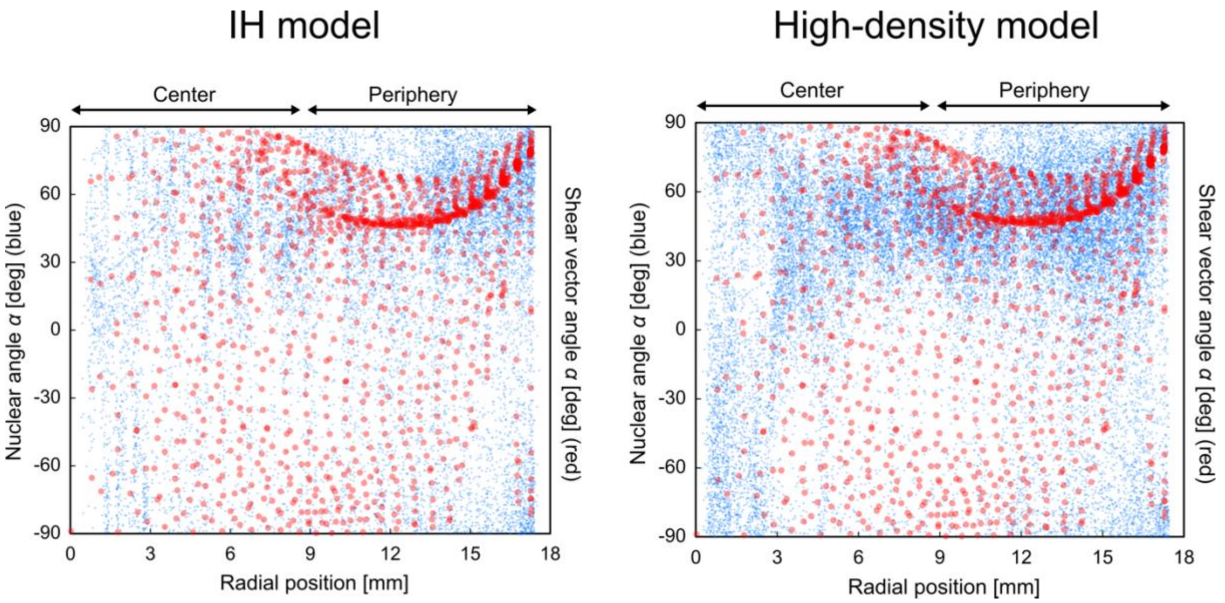

Figure S6 - ECs align in direction of the flow in both IH model and High-density model on s-SMCs. To correlate EC alignment with the direction of flow, low-density cocultures (IH model) and high-density cocultures (high-density model), were prepared according to protocol illustrated in **Figure 1A** and **4**. Graph showing the distribution of EC nuclei orientation in blue (angle α, degrees°) from center to periphery of the well (radial position). Orientation of EC nuclei stained with ERG1 (EC specific nuclear marker) as function of the radial position were analyzed using a Matlab script. Angle α is defined as indicated in **Figure S1E**. Red dots represent the angle α of shear vectors that were previously published [40] and kindly provided by the author. Blue dots that co-colocalize with red dots represent cells that are aligned with the flow. The vertical white patterns in the blue dots are an artifact derived from image stitching.

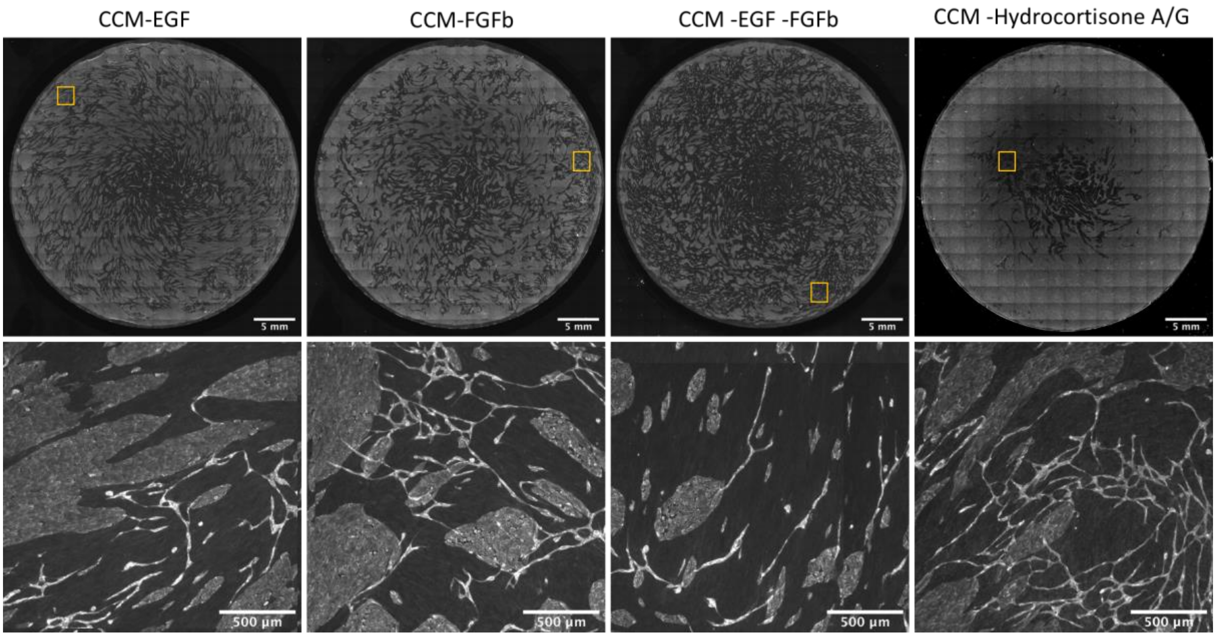

Figure S7 - EC networks form independently of any culture medium component. ECs were seeded on top of s-SMCs at low density and cultured for 3 days on the orbital shaker on coculture medium **(CCM, Table 1)**, without either EGF, FGFb, both EGF and FGFb, or both Hydrocortisone and Amphotericin/Gentamycin (A/G), but otherwise following the protocol shown in **Figure 1A** (IH model). EC networks formed in all conditions, but their prevalence was lower in CCM, without both EGF and FGFb. In all these conditions 2% FBS was present. Widefield fluorescence images showing VE-cadherin staining. Top images show the whole well as a large stitched mosaic. Scale bar 5mm. Below are shown the zoomed-in images of the respective overviews in the regions marked with a yellow square. Scale bar 500µm.

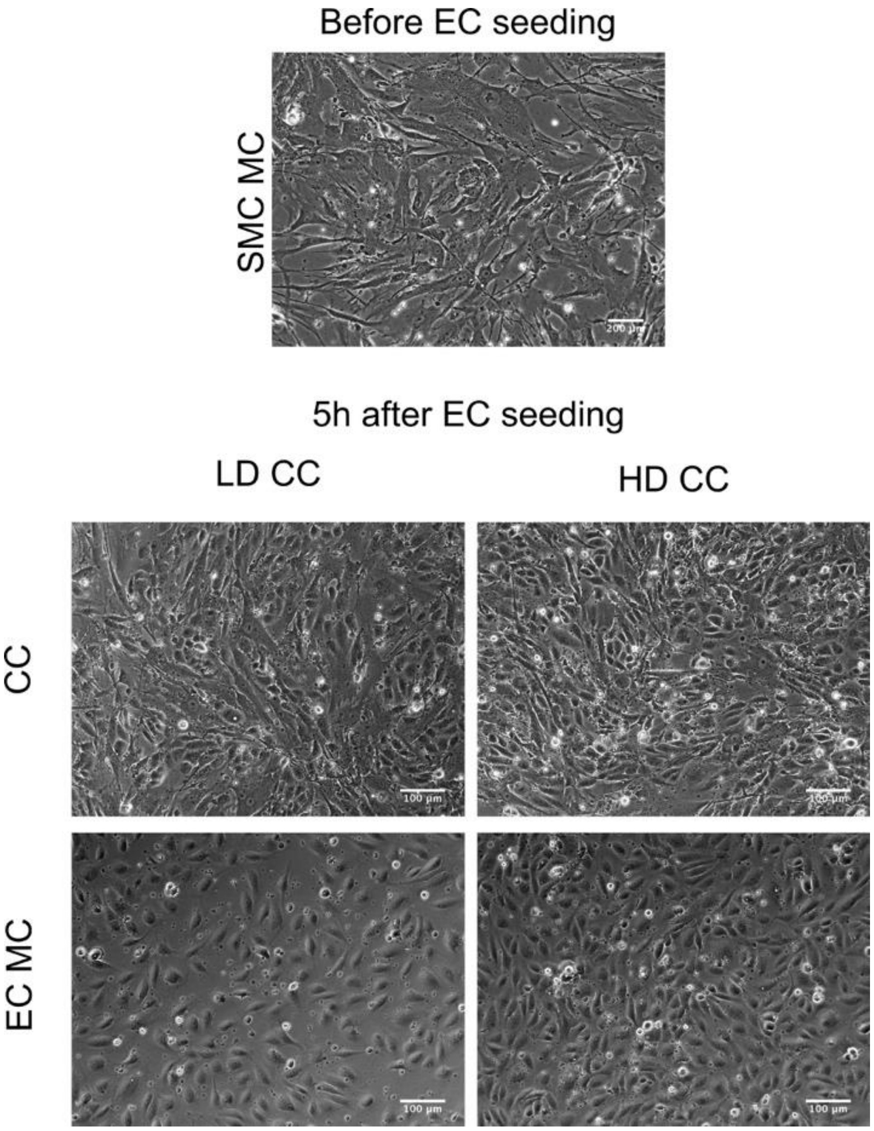

Figure S8 – High and low seeding density of ECs in mono- and cocultures. To compare the initial EC density in the IH **(Figure 1A, 1C, 2 and 3)** vs high-density model (**Figure 4**), phase-contrast images were acquired before and 5h after seeding ECs. A TPP 12-well plate with plastic bottom coated with 0.2% gelatin was used. **(On top)** s-SMCs were grown to confluency in SmGM medium and imaged with phase contrast in a Zeiss light microscope before addition of ECs. Scale Bar 200 µm. **(Below)** Thereafter, ECs were seeded at either 8x10^4 cells/cm^2 (low density, LD CC) or 1.5x10^5 cells/cm^2 (high density, HD CC). For seeding, MCDB131 medium (Invitrogen) containing 3.3% human serum, 1M Hepes, 1x Glutamax and 1x ITS (insulin-selenium-transferrin) was used. Cocultures (CC) and EC monocultures (EC MC) were imaged

5h after seeding with phase contrast using a Zeiss light microscope with a 10x objective. Scale bar 100µm.

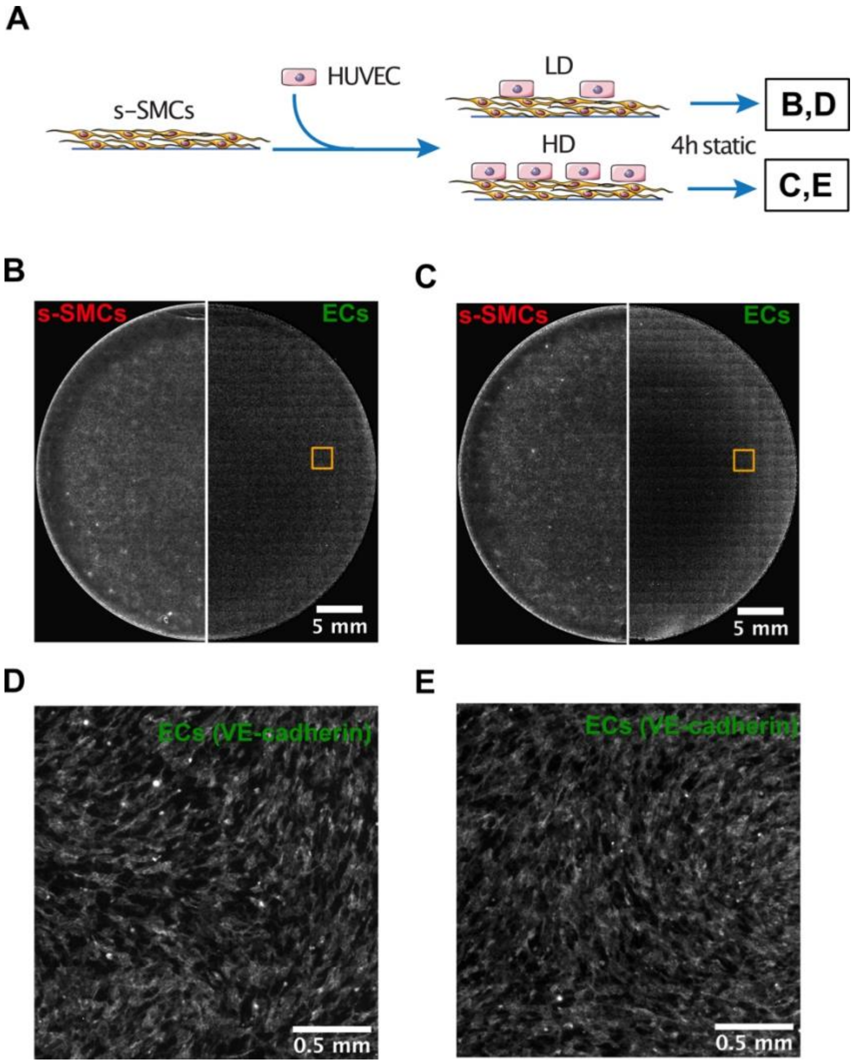

Figure S9 – Different EC seeding densities after 4h of coculture. To compare initial EC density in the intimal hyperplasia **(Figure 1A, 1C, 2 and 3)** vs high-density model (**Figure 4**), fluorescence images were acquired 4h after seeding ECs on top of s-SMCs. A glass-bottom 6-well plate coated with 0.2% gelatin was used. **(A)** Schematic cartoon with experimental protocol. ECs were seeded at high density ($1.5x10^5$ cells/cm^2, high-density model) or low density ($8x10^4$ cells/cm^2, IH model) on top of synthetic SMCs. **(B-E)** Fluorescence widefield image of the whole well 4h after seeding HUVECs at either **(B, D)** high density (HD) or **(C, E)** low density (LD). **(B-C)** Stitched mosaics of whole wells, depicting SMC labelled with cell tracker on the left and ECs immunostained with VE-cadherin on the right. Scale bar 5 mm. **(D-**

E) zoomed-in images of VE-cadherin staining in the regions marked with yellow squares. Scale bar 0.5 mm.

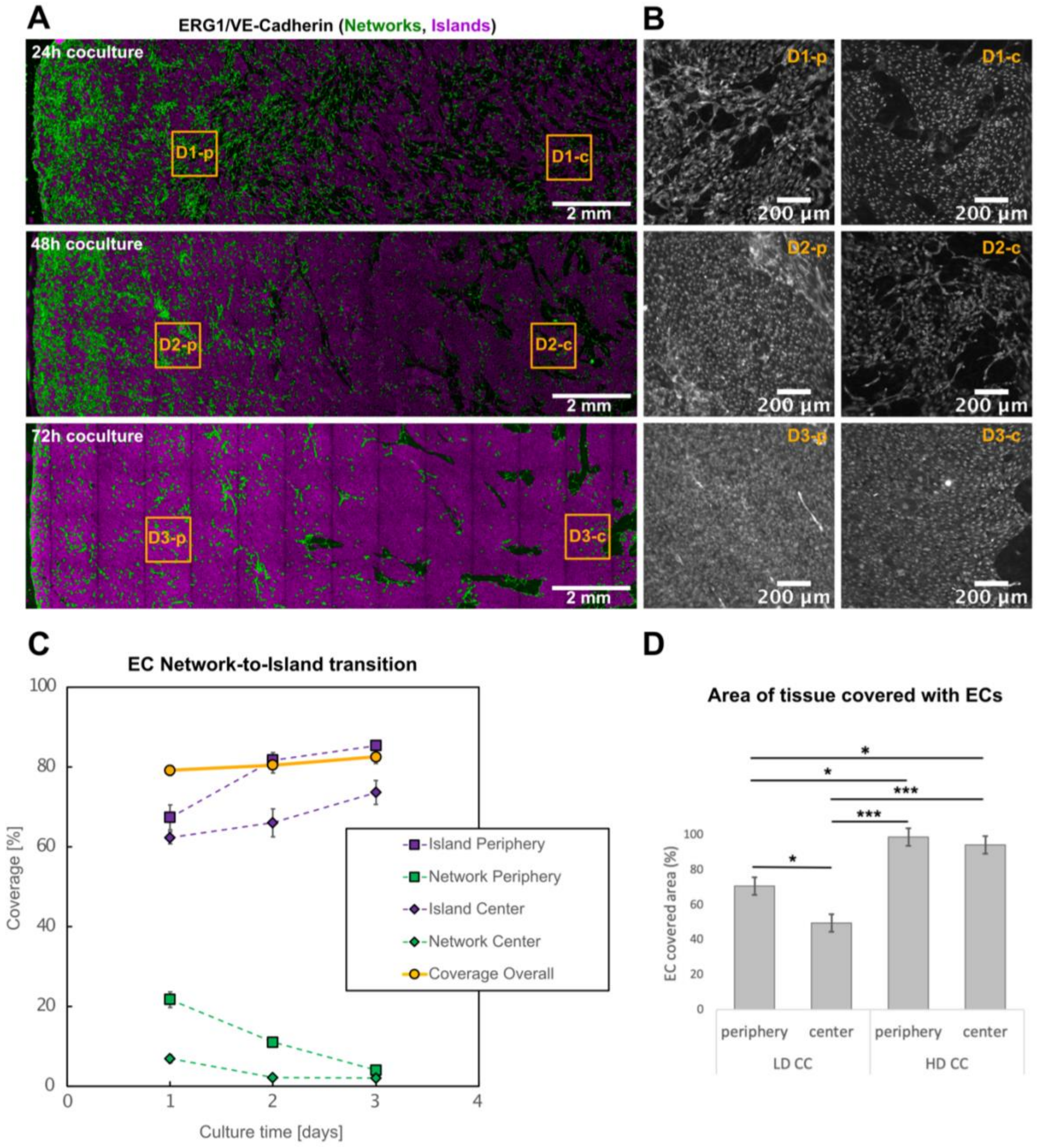

size

Figure S10 – Morphogenesis of endothelium on top of s-SMC after ECs were seeded at high density. The high-density model was prepared as described in **Figure 4**. **(A)** Widefield fluorescence images show a fraction of the tissue, from border to the center of the well, after 24h, 48h and 72h of coculture with s-SMCs on the orbital shaker. Each image belongs to an

independent sample. Double Immunostaining for EC nuclei (ERG1) and cell-cell junctions (VE-cadherin) using the same secondary antibody. s-SMCs are present, but not stained here to better visualization. Texture analysis allows to distinguish networks (green) from islands (purple). Scale bar 2mm. **(B)** Zoomed-in images of regions marked with yellow box in (A), showing double immunostaining for ERG1 and VE-cadherin. Day = D; periphery = p; center = c. Scale bar 200µm **(C)** Quantification of percentage of area covered with EC networks (green), islands (purple) in center end periphery regions of the well, and the overall percentage of area covered by ECs in the whole well (yellow). Analysis was done using different regions of interest from the images in Figure (A), therefore showing local variance. **(D)** Area covered by ECs in either the IH model, where ECs were seeded at low density (LD CC), or in the high-density model (HD CC), after 3 days on the orbital shaker in coculture medium (CCM, **Table 1**) as described in **Figure 1A and 4.** Total area covered with ECs was calculated using the large mosaic images showing VE-cadherin staining in the whole well or only half of the well (as in Figure 1A and C). VE-cadherin fluorescence signal was blurred and binarized for area calculation using Fiji software. Percentage of tissue covered with ECs was calculated based on the total area of the tissue, using Excel. Periphery and center regions of the well were compared. For statistics, T-test was performed (*** $p<0.0001$, ** $p<0.001$, *$p<0.01$).

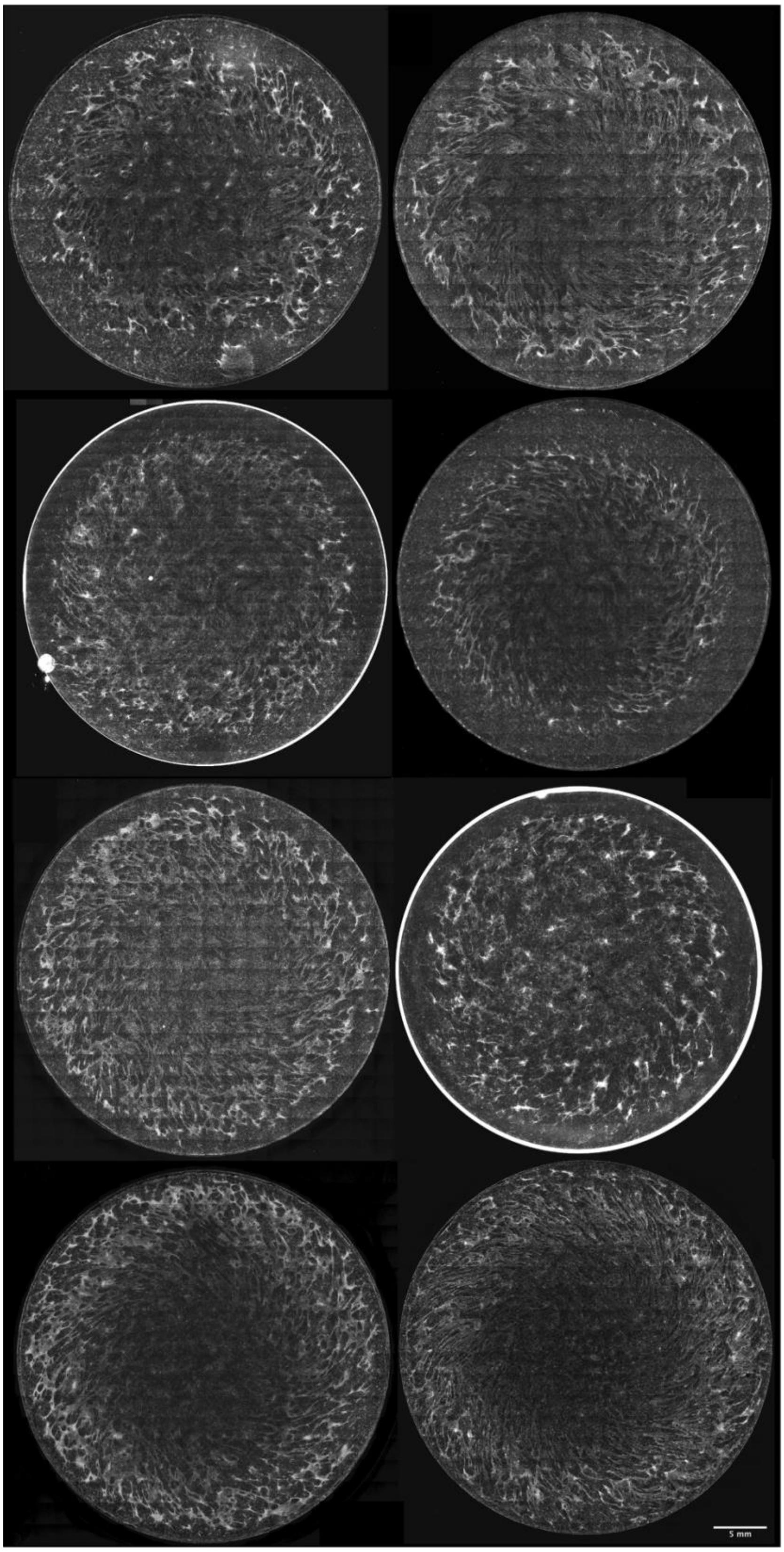

Figure S11 – Examples of SMC layers forming corrugated structures that resemble intimal thickening. The IH model was produced as described in **Figure 1A** and exposed to shear for 3 days on the orbital shaker using CCM **(Table 1)**. Images acquired by widefield

fluorescence microscopy show the whole well as a large stitched mosaic, with s-SMCs stained with cell tracker. Each image shows a separate sample prepared in different days. To simplify visualization ECs are not shown. The rectangular grid patterns result from stitching. Scale bar 5 mm.

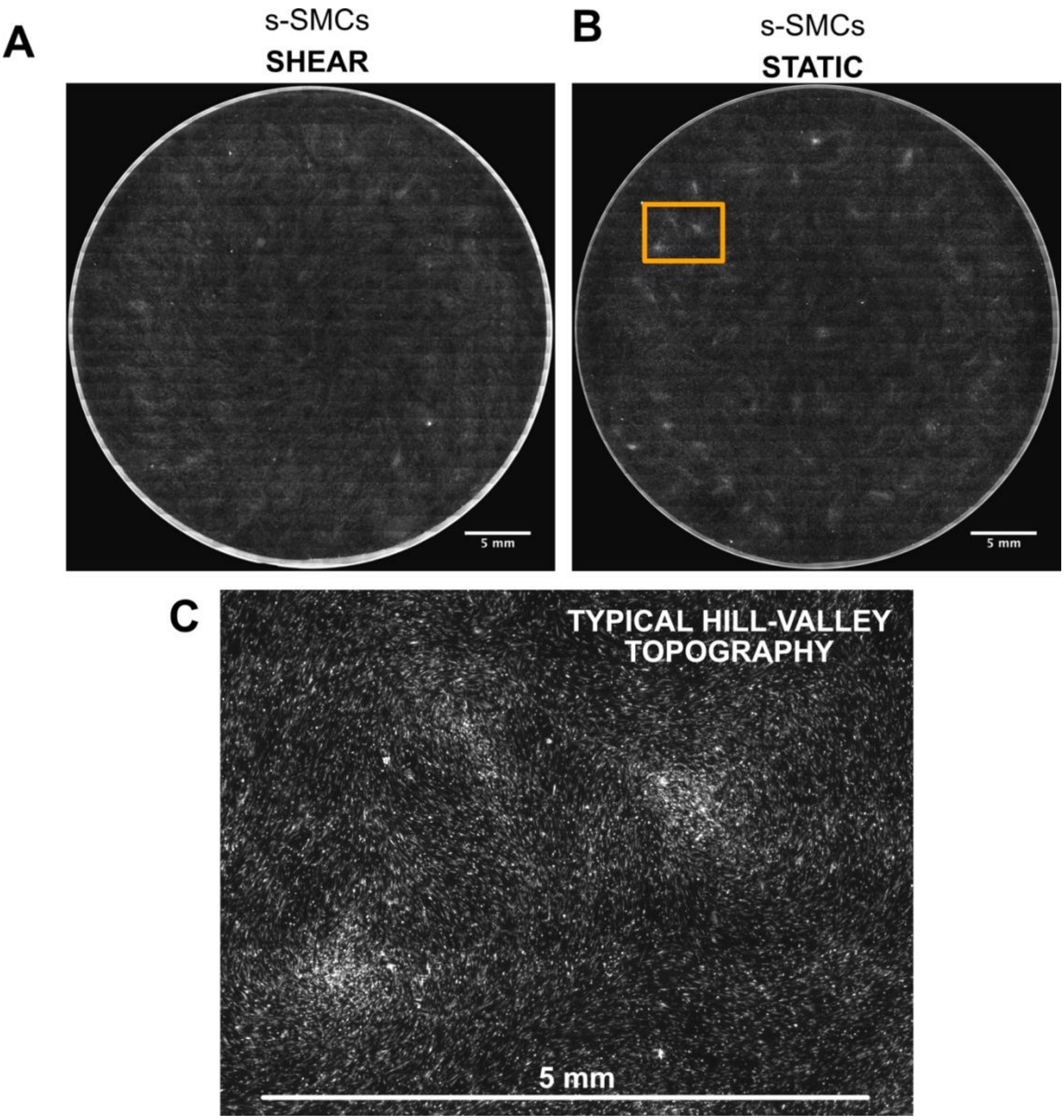

Figure S12 - SMCs typical "hill-valley" topography in monocultures. To show that the observed corrugated structures (**Figure 1C, 3A-C and S11**) are different from the hill-valley topography that SMCs typically show in a monoculture, human aortic primary s-SMCs were

grown until confluency in static conditions in SmGM **(Table 1)**. Thereafter, s-SMCs were transferred to m200 medium **(Table 1)** and grown under shear (A) or static (B) conditions for 3 days. Cells were labelled with cell tracker and imaged in a light microscope. **(A-B)** Large stitched mosaic showing the whole well. Scale bar 5 mm. **(C)** Zoomed-in image of yellow square in (B). Typical hill-valley topography is characterized by hills of cells, often organized in spirals. Scale bar 5 mm.

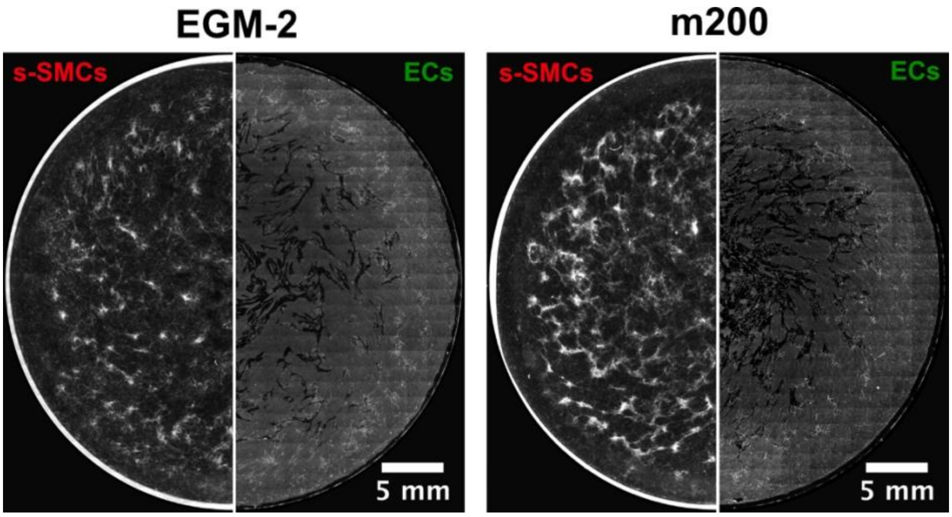

Figure S13 – IH model using different coculture media. Intimal hyperplasia model, where ECs were seeded at low density on s-SMCs, according to the protocol sketched in **Figure 1A**. Black and white images acquired by widefield fluorescence microscopy show the whole well as a large mosaic. On the left side: s-SMCs stained with cell tracker. On the right side: ECs through VE-cadherin immunostaining. Both culture media are commercially available endothelial growth media: EGM-2 (Lonza) and m200 (Invitrogen). See **Table 1** for complete media formulas. Scale bar 5 mm.

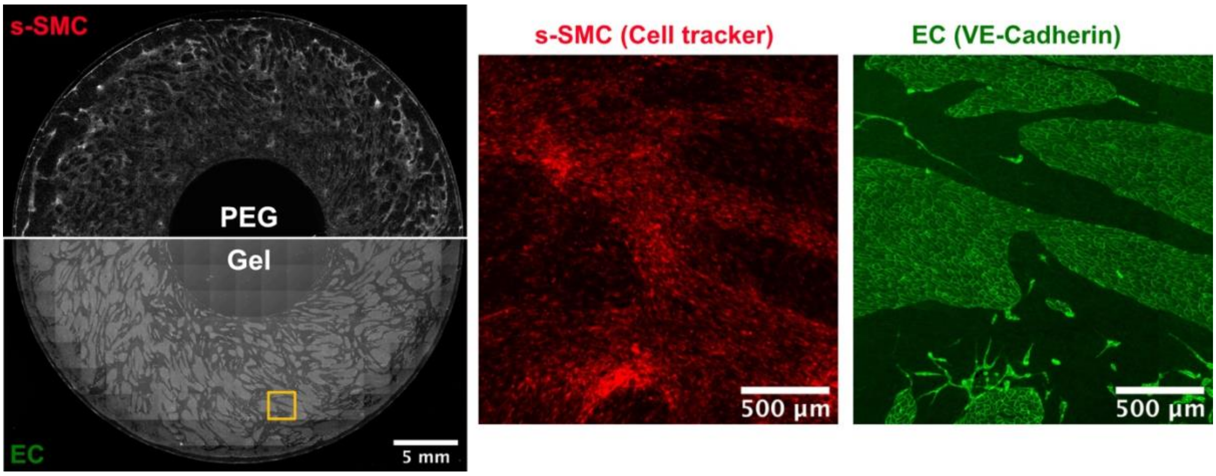

Figure S14 - Corrugated structures are also observed in cocultures where the center of the well was rendered non-adherent to cells. To verify whether cells from the central region of the well, where the flow is disturbed, were responsible for the formation of SMC corrugated structures at the periphery, the center of the well was rendered non-adherent to cells. A drop of 3-trimethoxysilyl propyl methacrylate (TMSPMA) was added in the center of the well and incubated in the oven at 80°C for 30min, followed by addition of a drop of PEG and UV light for 30 seconds. Thereafter, the whole well surface was coated with 0.2% gelatin. Coculture of low density seeded ECs on top of s-SMCs for 3 days in CCM on the orbital shaker (IH model), as described in **Figure 1A.** Black and white image on the left, acquired by widefield fluorescence microscopy, shows a stitched mosaic of the whole well. Top: synthetic SMCs (s-SMC) stained with cell tracker. Bottom: ECs stained with anti-VE-cadherin antibody. Scale bar 5 mm. On the right, zoomed-in images of the yellow square show s-SMCs stained with cell tracker (red) and ECs stained with VE-cadherin (green). Scale bar 500µm.

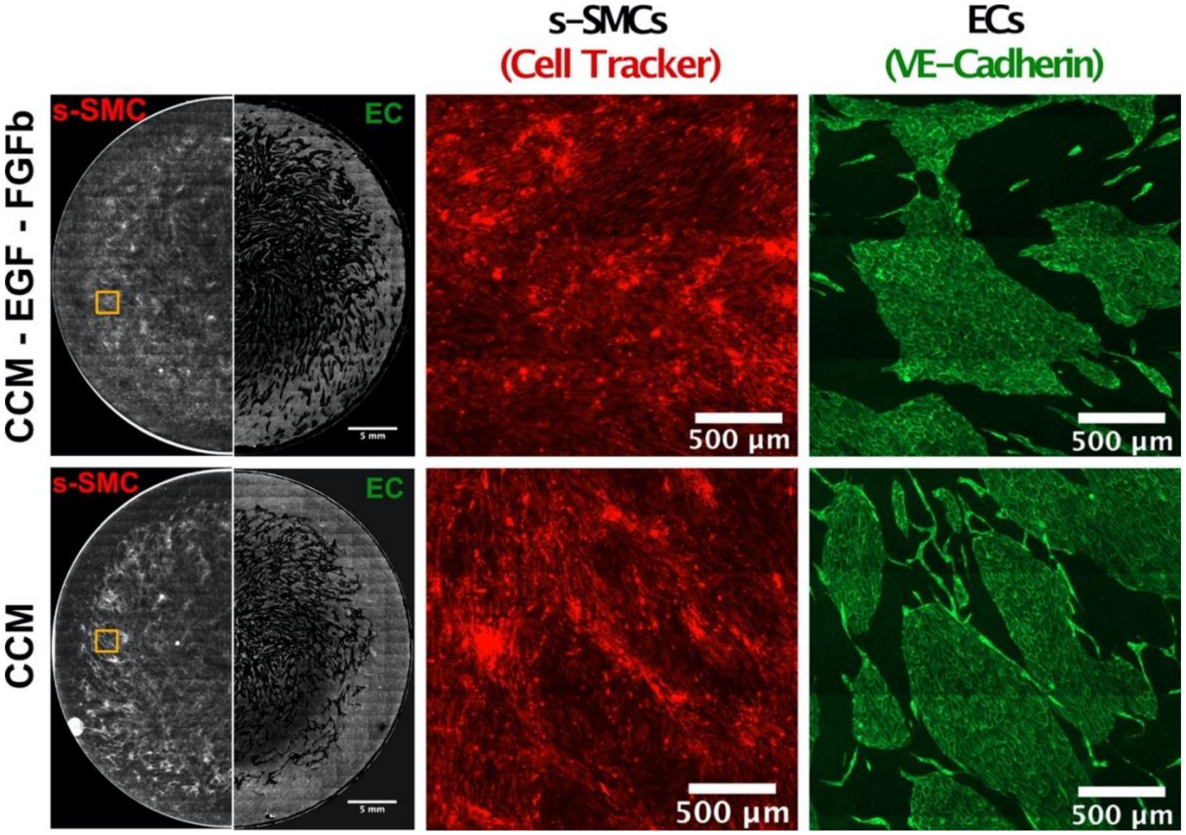

Figure S15 – Removal of EGF and FGFb from the coculture medium impairs regeneration of EC layer and inhibits formation of corrugated structures. To investigate the role of growth factors in the formation of SMC corrugated structures, ECs were seeded on top of s-SMCs at low density and cultured for 3 days on an orbital shaker in either coculture medium (CCM) or CCM without the addition of EGF and FGFb (CCM-EGF-FGFb). See **Table 1** for complete media formulas. The black/white images acquired by widefield fluorescence microscopy show the whole well as a large stitched mosaic, with s-SMCs stained with cell tracker on the left half and ECs stained with VE-cadherin immunostaining on the right half. Scale bar 5mm. Zoomed-in images of yellow squares show s-SMCs (red), ECs (green). Scale bar 500µm.

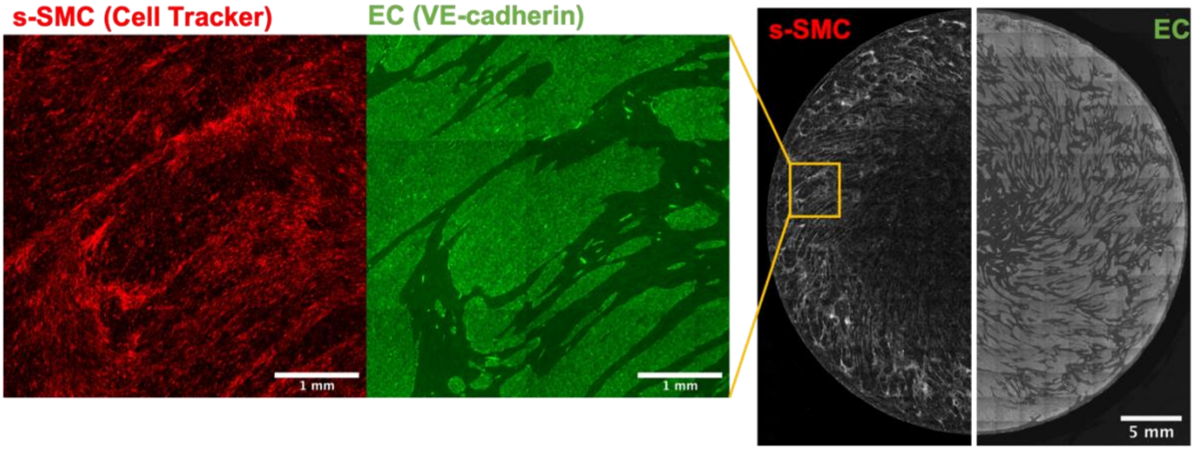

Figure S16 – Example of corrugated structures forming in region without EC networks. Corrugated structures are more pronounced in regions of EC networks **(Figure 3E).** However, to prove that EC networks are not necessary for SMC corrugated structures to form, we provide an example here of a region with corrugated structures, but no EC networks. ECs were seeded on top of s-SMCs at low density (IH Model) and cultured for 3 days on the orbital shaker on CCM, as described in **Figure 1A**. Black and white image acquired by widefield fluorescence microscopy shows a stitched mosaic of the whole well. Left: synthetic SMCs (s-SMC) stained with cell tracker. Right: ECs stained with anti-VE-cadherin antibody. Scale bar 5 mm. On the left, zoomed-in images of the yellow square show s-SMCs stained with cell tracker (red) and ECs stained with VE-cadherin (green). Scale bar 1mm.

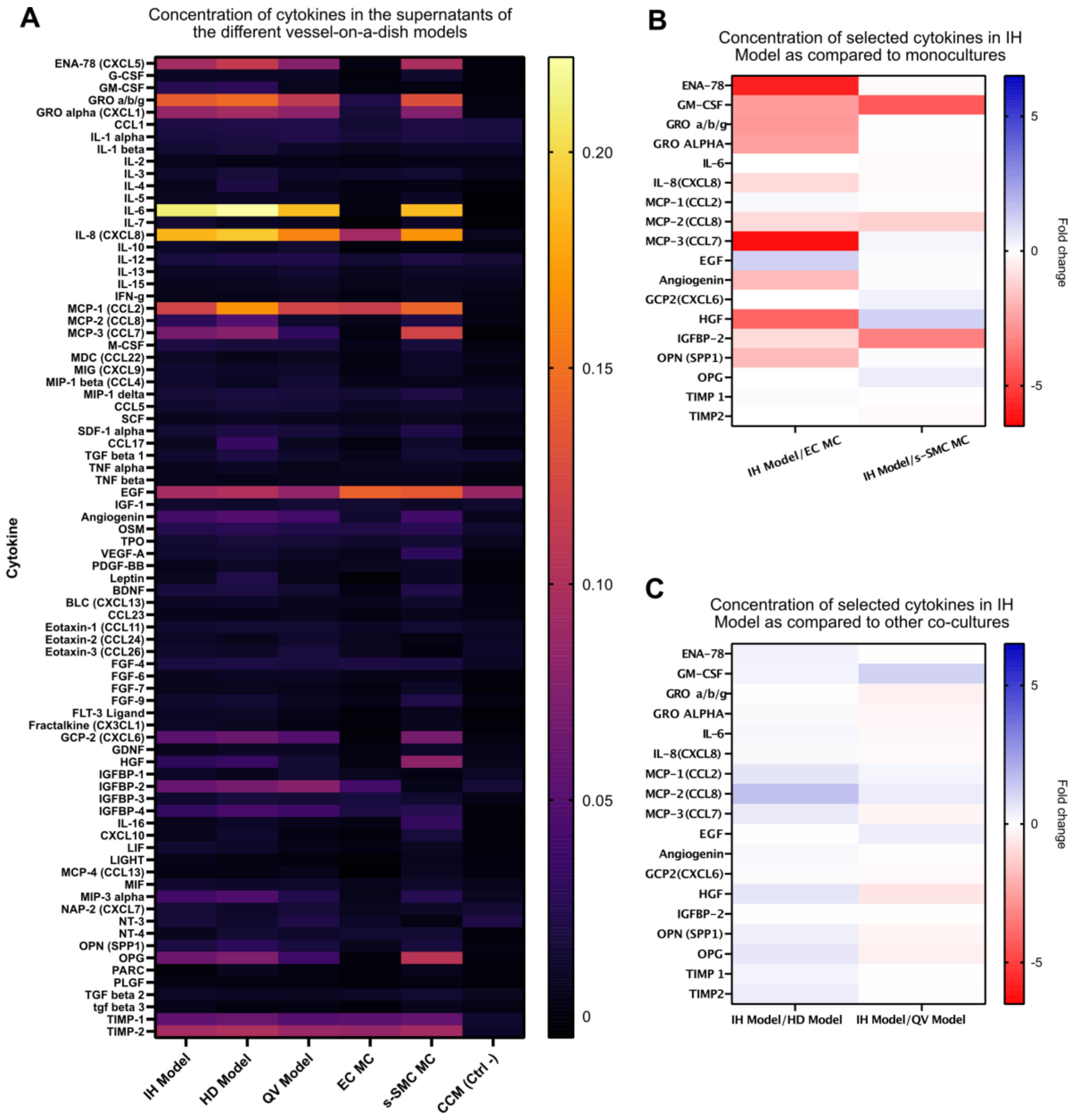

Figure S17 - In coculture, pro-inflammatory cytokines are secreted. A cytokine array was performed with the supernatants from cocultures at day 3. **(A)** The absolute concentration of cytokines in the supernatant is compared between the different models and monocultures. Coculture medium (CCM, Table 1) was used as negative control. Intimal hyperplasia model (IH Model), high-density model (HD Model), quiescent vasculature model (QV Model), EC monoculture (EC MC) and s-SMC monoculture (s-SMC MC). **(B-C)** The concentration of the most relevant cytokines in IH model were compared with **(B)** monocultures and with **(C)** HD model and QV model. The graphs show the fold change. Cytokines that are present at higher

concentrations in IH model as compared with the other models are shown in red, and lower in blue.

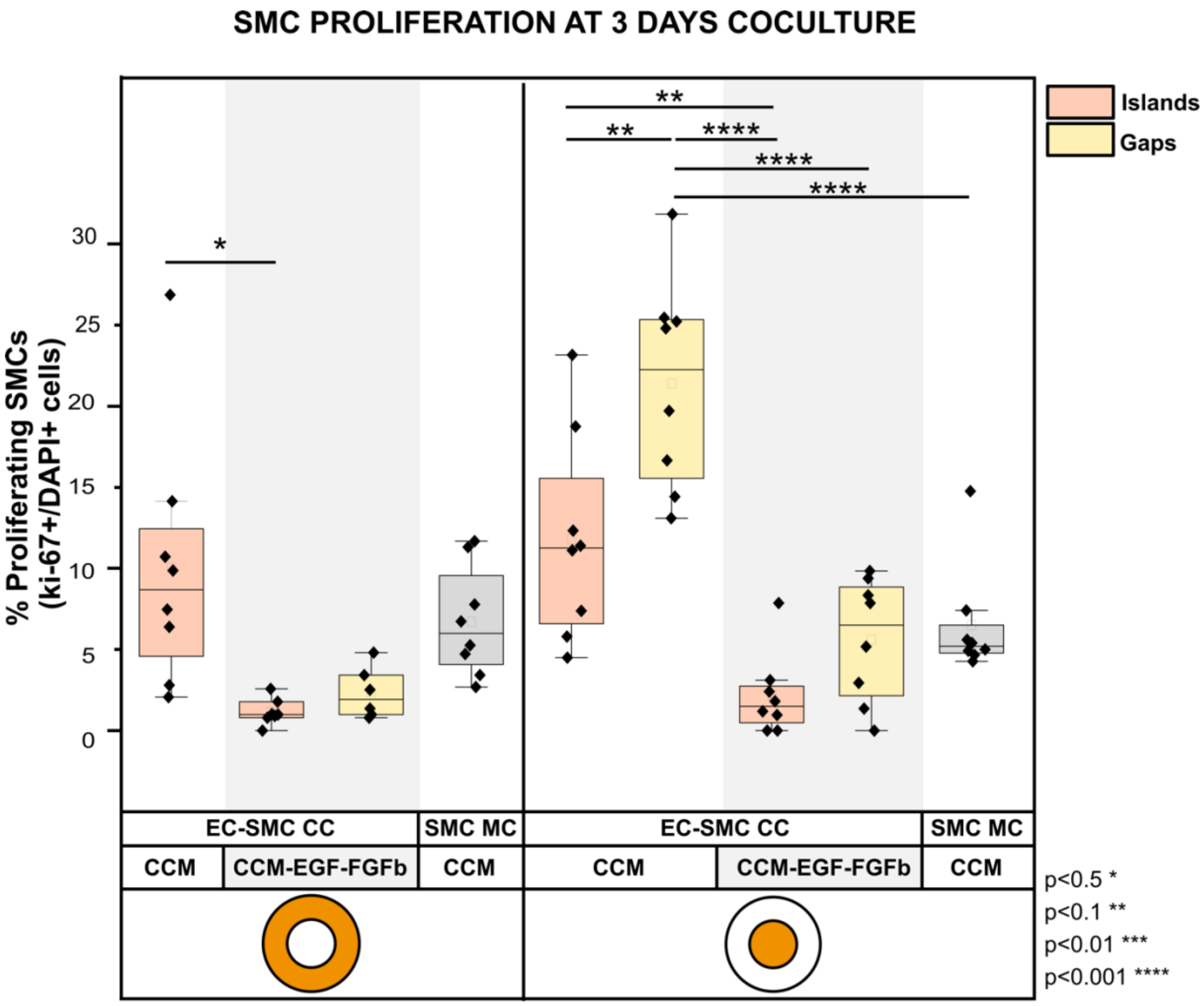

Figure S18 – Number of proliferating SMCs is reduced in the absence of EGF and FGFb. Graph showing the percentage of proliferating SMCs after 3 days of coculture with ECs at low-density under shear (EC-SMC CC = IH Model). Either complete coculture medium (CCM) or CCM without both EGF and FGFb was used (CCM-EGF-FGFb) (see **Table 1**). The different regions of the tissue (EC islands and Gaps) in both center and periphery regions of the well were compared. As a control, s-SMC monocultures (SMC MC) were cultured for 3 days under shear in CCM. Proliferative cells were immunostained with anti-ki-67 antibody, a proliferative marker, and all nuclei were stained with DAPI. To selectively count SMC nuclei, ERG+ cells (EC nuclei) were excluded. Nuclei were counted using a script in Fiji/ImageJ.

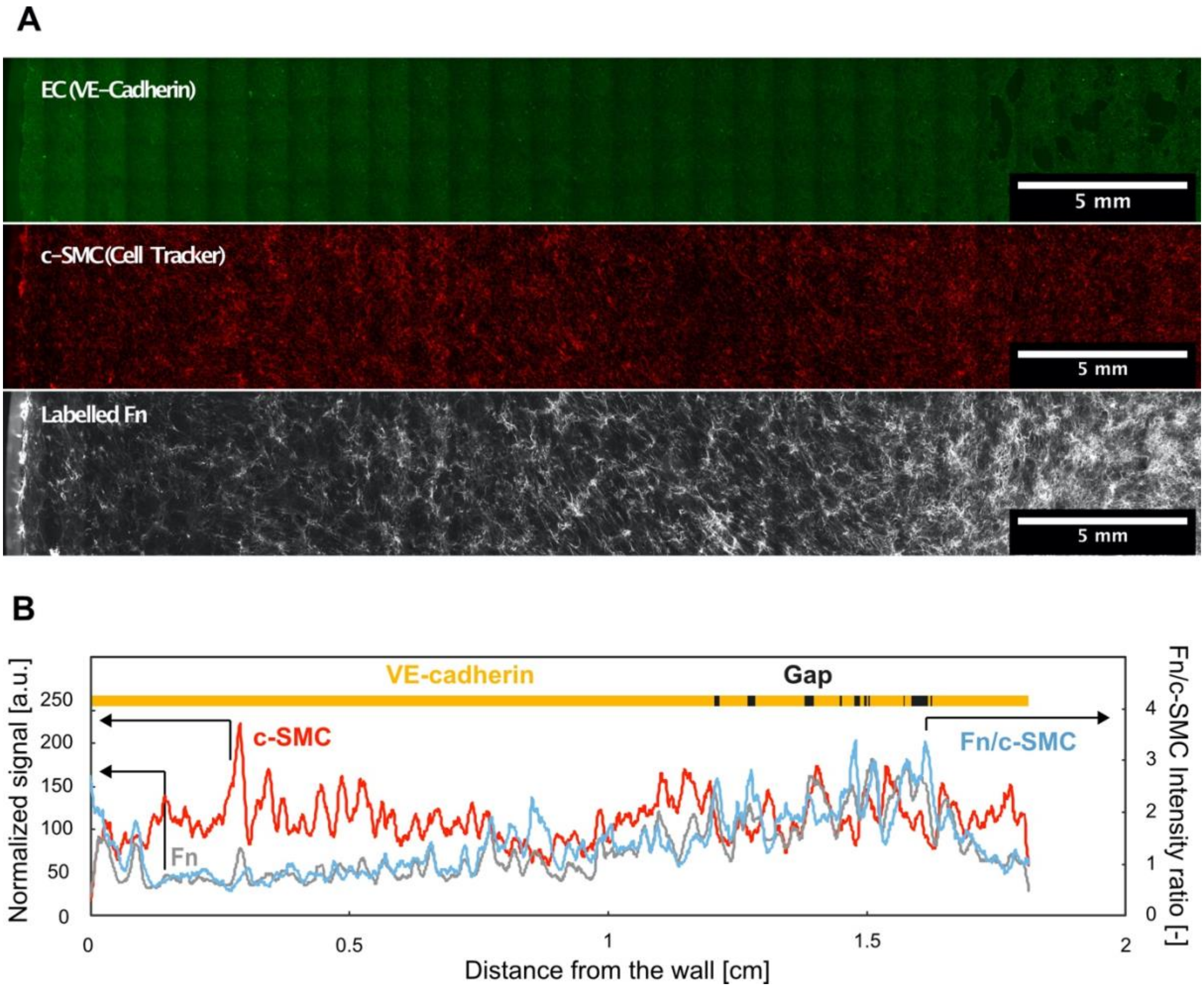

Figure S19 – In cocultures with c-SMCs, confluent endothelium inhibits formation of corrugated structures and significantly reduces incorporation of plasma fibronectin into the ECM. (A) Wide-field fluorescence image of a coculture (quiescent vasculature model) with c-SMC, after 3 days on the orbital shaker. Periphery of the well on the left and center of well on the right. Green - VE-cadherin; Gray - fibronectin labelled with Alexa 647; Red - SMC labelled with cell tracker. **(B)** Graph showing intensity plot of cell tracker (SMCs, red) and labelled plasma fibronectin (Fn, gray), binarized signal of VE-cadherin immunostaining (EC islands - yellow, EC networks - green, Gaps - black) and fibronectin intensity normalized by cell tracker intensity (Fn/SMC) from periphery to center of the well.

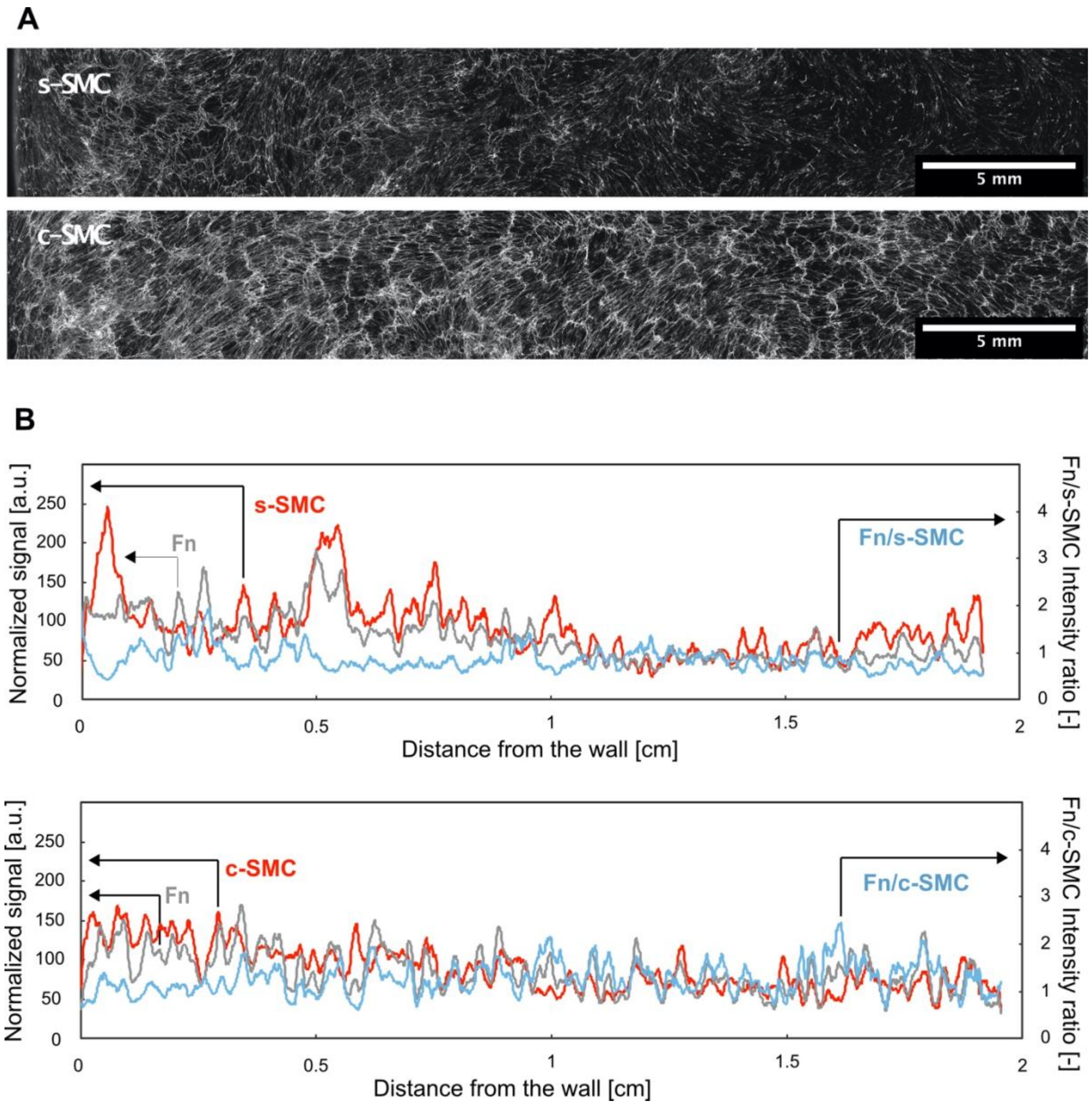

Figure S20 – Incorporation of supplemented labelled plasma Fibronectin into ECM is homogeneous in s-SMC and c-SMC monocultures. (A) Wide-field fluorescence image of a SMC monocultures with either s-SMCs or c-SMC, after 3 days on the orbital shaker**.** Periphery of the well on the left and center of well on the right. Gray - fibronectin labelled with Alexa 647. **(B)** Graph showing intensity plot of cell tracker (s-SMCs or c-SMC, red) and labelled plasma fibronectin (Fn, gray) and fibronectin intensity normalized by cell tracker intensity (Fn/s-SMC or Fn/c-SMC, blue) from periphery to center of the well. Plot of a s-SMC monoculture on top and of a c-SMC monoculture below.

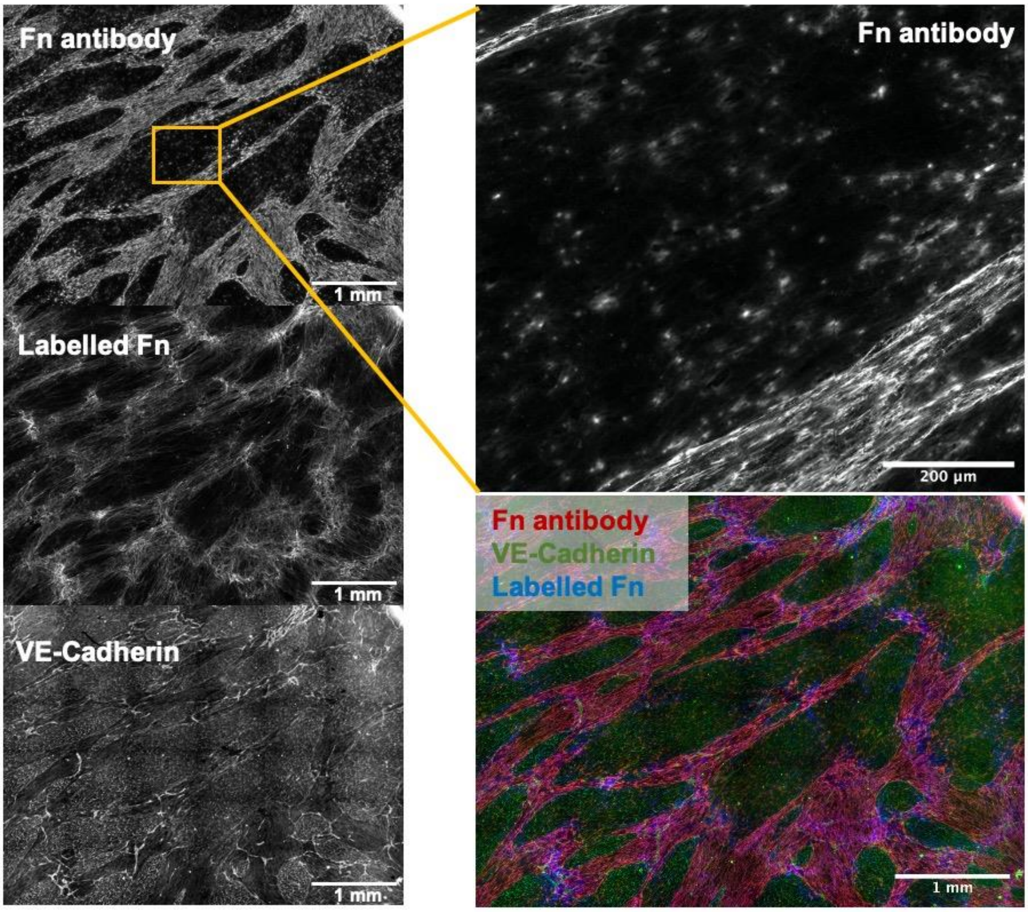

Figure S21 – Polyclonal anti-fibronectin antibody access to the SMCs-assembled ECM is mostly blocked by the confluent endothelium. Wide-field fluorescence image of a low-density coculture with s-SMC (IH model, prepared as described in **Figure 1A**), after 3 days on the orbital shaker. Image was taken at the periphery of the well. On the top right: zoomed-in image of yellow rectangle showing fibronectin (Fn) antibody staining. Scale bar 200µm. On the bottom right: merged image showing total fibronectin detected with goat polyclonal fibronectin antibody that binds to C-20 terminus (red), VE-cadherin (green) and supplemented plasma fibronectin labelled with Alexa647 (blue). Scale bar 1mm.

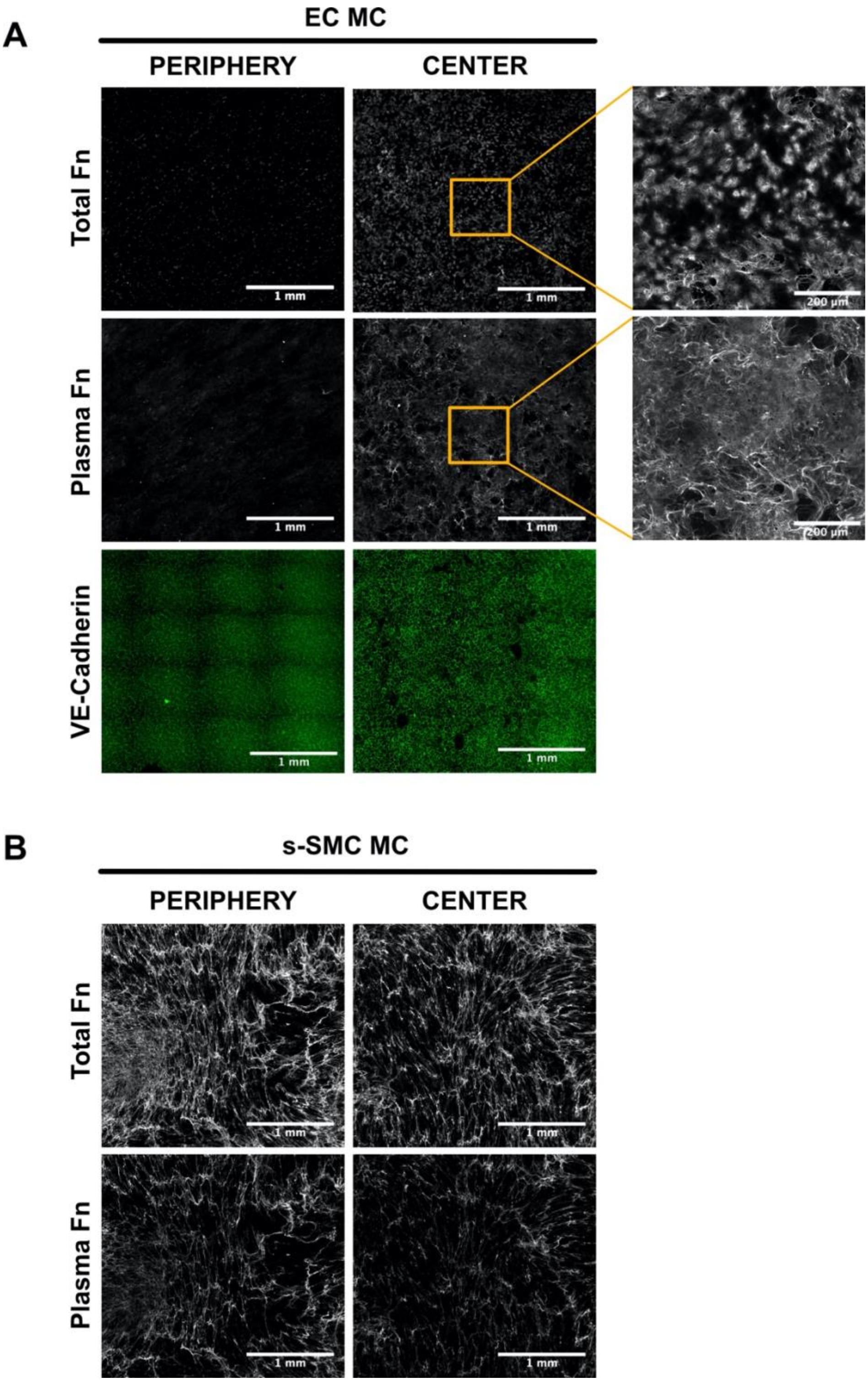

Figure S22 – Anti-fibronectin polyclonal antibody and supplemented plasma fibronectin in EC and s-SMC monocultures. (A) Wide-field fluorescence image of EC monocultures (EC MC), after 3 days on the orbital shaker in coculture medium (CCM**, Table 1**). Scale bar 1mm. On the right: zoomed-in images of yellow squares. Scale bar 200µm. **(B)** Wide-field

fluorescence image of s-SMC monocultures (s-SMC MC), after 3 days on the orbital shaker in SmGM (**Table 1**). Scale bar 1mm. **(A-B)** Images were taken at the periphery and center of the well. Total fibronectin detected by immunostaining with goat polyclonal fibronectin antibody that binds to C-20 terminus (Total Fn, grey), supplemented plasma fibronectin labelled with Alexa647 (Plasma Fn, grey) and VE-cadherin immunostaining (green).

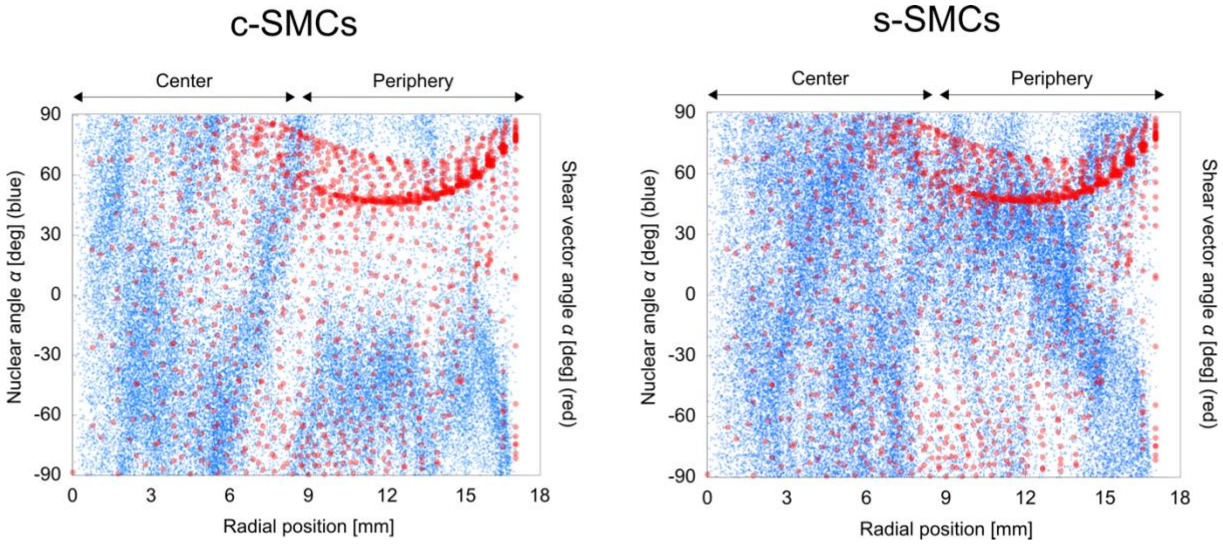

Figure S23 – SMCs do not align with the flow. Contractile SMC (c-SMCs) and synthetic SMC (s-SMCs) monocultures cultured in coculture medium (CCM, **Table 1**) for 3 days either on the orbital shaker or under static conditions were compared. Orientation of SMC nuclei stained with DAPI (blue dots) as function of the radial position were analyzed using a Matlab script. Angle α is defined as indicated in **Figure S1E**. Red dots represent the angle α of shear vectors that were previously published[40] and kindly provided by the author. Blue dots that co-colocalize with red dots represent cells that are aligned with the flow. The vertical white patterns in the blue dots are an artifact derived from image stitching. Although SMC alignment did not match with vectors of shear in none of the conditions, c-SMCs cells in the periphery seem to preferentially align perpendicular to the shear (-60°).

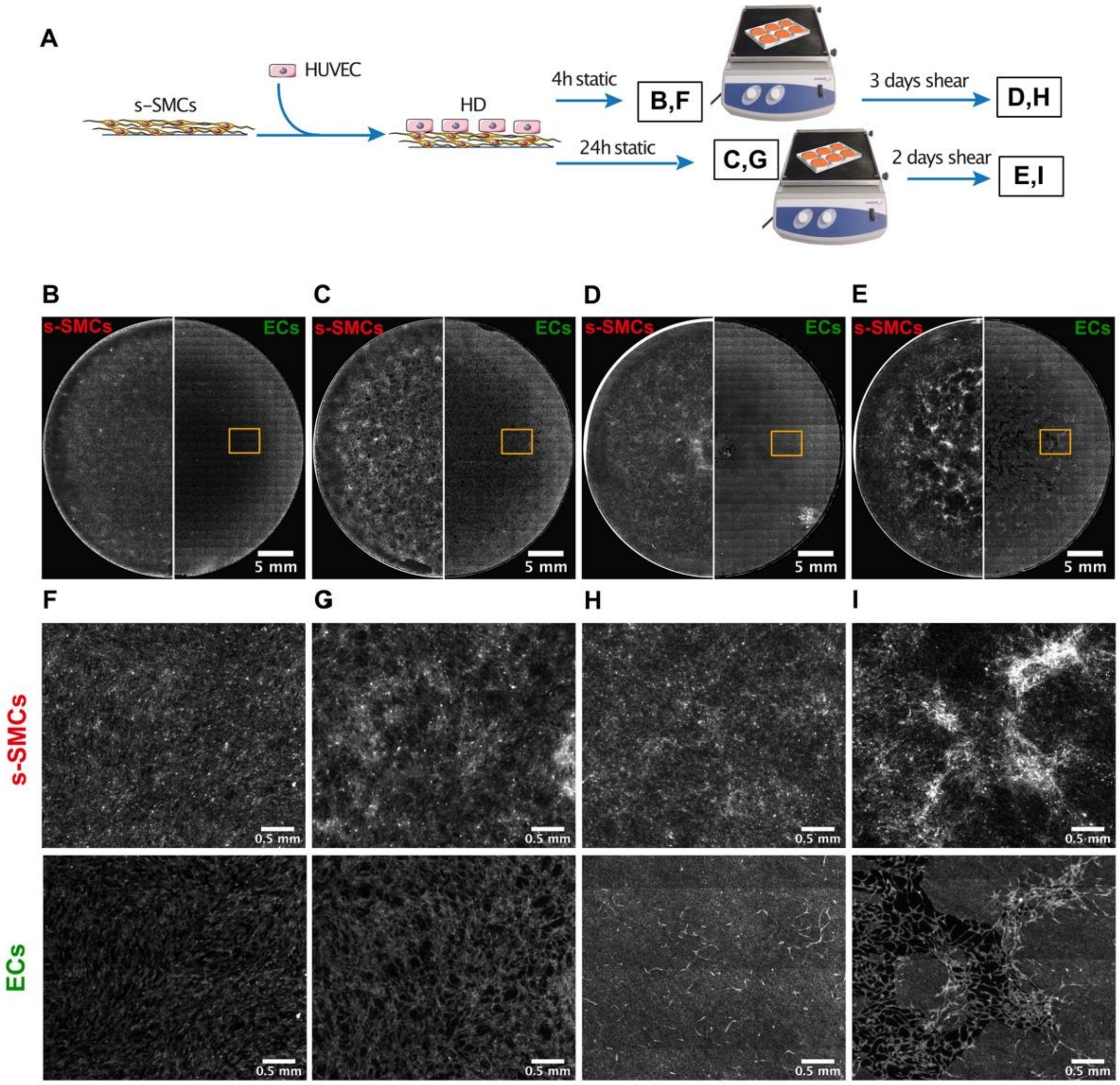

Figure S24 - The timing of placing cocultures on the orbital shaker determines whether EC monolayers become confluent or not. To investigate the effect of shear in the initial phase of establishing a stable coculture, high-density (HD) cocultures were placed on the orbital shaker either 4h or 24h after initiation of coculture. **(A)** Schematic cartoon with the experimental protocol to create the high-density model. Human umbilical vein endothelial cells (HUVEC) were seeded at high density ($1.5x10^5$ cells/cm^2) on top of human aortic smooth muscle cells with synthetic phenotype (s-SMCs) and cultured in static conditions (B, F) for 4h or (C, G) 24h in coculture medium (CCM, **Table 1**). Additionally, ECs were seeded at high density on top of s-SMCs and (D, H) cultured in static conditions for 4h and on the orbital

shaker for additional 3 days, or (E, I) 24h in static conditions followed by 2 days on the orbital shaker, both in m200 medium **(Table 1)**. **(B-E)** Stitched mosaics of whole wells depicting SMC labelled with cell tracker on the left and ECs immunostained with VE-cadherin on the right. Scale bar 5 mm. **(F-I)** Zoomed-in images of both cell tracker (top) and VE-cadherin channels marked with an orange square in the respective whole-well overviews. Scale bar 0.5mm.

2. SUPPLEMENTARY METHODS

2.1. Comparison of VE-cadherin, Fibronectin and SMC cross-sectional profiles

Stitched images with 3 channels were used (channel 1: cell tracker stained SMCs; channel 2: ERG1 and VE-cadherin double staining, i.e. two different primary antibodies targeted with the same secondary antibody; channel 3: Fibronectin). ERG1/VE-cadherin images were blurred and binarized by thresholding. The positive and negative regions in the binarized ERG1/VE-cadherin image were identified as “islands” and “gaps”, respectively. In the image from the IH model, regions of EC networks and island regions were manually identified. These ERG1/VE-cadherin binarized images indicated gaps, islands and EC networks, and the corresponding raw images of Fibronectin (Fn) and SMC were compressed to low-resolution (around 240 x 1960 pixels). The Fn and SMC images were then processed using a mean filter kernel of 15 x 15 pixels window size to remove signal fluctuations in 10 µm scales and to better visualize the large-scale trends. The contrast of each image was linearly adjusted so that its maximum pixel intensity becomes 255 in the end. Using these classified and processed images of the three channels, the cross-sectional profiles at arbitrary positions were created. Note that these processed images were only used to create cross-sectional profiles with the normalized range and were not used for quantitative analysis. See these results in **Figure 3F, 4F, S19B** and **S20B.**

2.2. Orientation of Cell Nuclei

For SMC and EC monocultures, DAPI images were utilized. For cocultures, images with ERG1 and VE-cadherin double staining (i.e. two different primary antibodies targeted with the same secondary antibody) and DAPI images were utilized. DAPI and ERG1/VE-cadherin images were blurred and binarized by thresholding. The binarized images in pairs were then processed using a custom script written in MATLAB (2016b, Mathworks, Natick, MA, USA). Briefly, the connectivity of the signals in the DAPI image was analyzed to distinguish each nucleus. To select ERG-1-positive nuclei and to remove the VE-cadherin signal, with each DAPI-positive nucleus the number of positive pixels in the corresponding area of the binarized ERG1 image were counted. Then, the ERG1-positive ratio was evaluated by dividing the number of ERG1-positive pixels by the total number of DAPI-positive pixels. In the end, a nucleus with an ERG-positive ratio higher and lower than 50% was recognized as that of ECs and SMCs, respectively. Thereafter, nuclear orientations were evaluated using a custom script written in MATLAB. First, the position of the dish center was detected by fitting a circle to the edge of the cell culture dish in the binarized nuclei images. Next, connectivity analysis was performed to distinguish each nucleus. Each nucleus was then fit to an ellipse. The angle between the long axis of the nucleus and the radial direction was evaluated as the nuclear angle α (as defined in **Figure S1E**). This angle was defined according to Salek et al. [40], so that the angle of shear vectors simulated in this publication can be compared with the orientation of cells in this study. See the corresponding results in **Figure S1D, S6** and **S23.**

2.3. Immunostainings and Microscopy

Tissues/cells were fixed with 4% paraformaldehyde for 15 min at room temperature (RT). If necessary, cells were permeabilized for 10 min with 0.1% Triton. Thereafter, cells were blocked with 1% BSA for 1 hour at RT and stained with primary antibodies in 1% BSA overnight at 4°C. After 3 washes with 1x PBS, samples were incubated with secondary antibody for 1h at

RT, followed by another 3 washes with 1x PBS and stored in 1x PBS at 4°C before imaging. See antibody details on **Table S1**. When indicated, cell nuclei were stained with DAPI (Invitrogen, D3571) for 10 minutes, 1:500 dilution in PBS.

**Primary Antibody**	**Dilution Primary Antibody**	**Secondary Antibody**	**Dilution Secondary Antibody**
Rabbit anti- VE-cadherin (Cell Signaling, 2500)	1:400-1:500	Goat anti-rabbit AlexaFluor-488 (Invitrogen A11034) OR donkey anti-rabbit 546 (Invitrogen A10040)	1:250
Mouse anti-αSMA (Sigma, a2547)	1:400	Goat anti-mouse AlexaFluor-647 (Abcam, ab150115)	1:250
Rabbit anti-Calponin (Abcam, ab46794)	1:250	Goat anti-rabbit AlexaFluor-647 (ThermoFisher, A21245)	1:250
Rabbit anti-S100A4 (Abcam, ab124805)	1:250	Goat anti-rabbit AlexaFluor-647 (ThermoFisher, A21245)	1:250
Mouse anti-Ki-67 (Novus Biologics, NBP2-22112)	1:1000	Donkey anti-mouse AlexaFluor-647 (Invitrogen 150107	1:250
Rabbit anti-ERG1 (Cell Signaling, 97249S)	1:500	Goat anti-rabbit AlexaFluor488 (Invitrogen, 11034)	1:250
Goat anti-Fibronectin (Santa Cruz, sc6953)	1:100	Donkey anti-goat AlexaFluor 647	1:250

Table S1 - Primary and secondary antibodies used for immunostaining and their respective dilutions.

Images were taken with either an Olympus FV1000 laser scanning confocal microscope using Olympus FV10-ASW version 03.01 software, Zeiss AxioObserver.Z1 using Zen Black software (ScopeM facility, ETH Zurich), ImageXpress micro (IXM) from Molecular Devices using Molecular Devices MetaXpress 5 Software (ScopeM facility, ETH Zurich), ZEISS AxioObserver ApoTome.2 inverted epifluorescence microscope using ZEN software

(laboratory of Prof. Zenobi-Wong, ETH Zurich) or Nikon Eclipse Ti2 epifluorescence microscope using Nikon NIS-Elements Advanced Research 5.02 software (laboratory of Prof. Simone Schürle-Finke, ETH Zurich). Only images within the same figure were imaged and edited with the same settings and can be directly compared.

2.4. Western Blotting

Cells were lysed with 100µl of lysis buffer, containing 20mM TRIS (pH 7.5), 150mM NaCl, 1mM EGTA, 1mM EDTA and 1% Triton-X-100 in 1x PBS. EDTA-free Protease Inhibitor Cocktail (Sigma Aldrich 11836170001; 1 tab diluted in 1ml of water, use at 1:50 dilution) and phosphatase inhibitors (Sigma-Aldrich, P5726-1ML, 1:100 dilution) were added. The lysate was transferred to an eppendorf tube and placed on an end-over spinning for 20min at 4°C. After centrifugation at 10600g for 10min, the supernatant was recovered and aliquoted for Micro BCA measurement and western blotting. The Micro BCATM Protein Assay Kit (ThermoScientific, #23235) was used to measure the total protein content of the sample. For protein separation by size, an equal amount of protein was loaded in each gel pocket. Electrophoresis was performed with 60V for 25min, then 1h 30min at 110V. The Mini-Protean Tetra Cell System from BioRad was used to transfer the protein to a membrane with pore size 0.2µm (Amersham Protran ,10600015), at 90V for 90 minutes. Afterwards, the membranes were blocked with 5% milk in 1xTBST (0.1% Tween) for 1 hour at room temperature. Primary antibodies were incubated overnight at 4°C, diluted in 5% milk at their respective dilutions (see antibody details on **Table S2**). After three washes with 1x TBST, membranes were incubated with secondary antibodies (1:10'000 in 5% milk) for 1h at room temperature followed by three washing steps with 1x TBST and one wash with 1x TBS. Table 3 shows the antibodies that were used for the western blot with the corresponding dilutions and molecular weight of the protein. Used secondary antibodies were either donkey anti-rabbit HRP conjugated (Jackson, 711-035-152) or donkey anti-mouse HRP conjugated (Jackson, 715-035-150), diluted 1:10'000

in 5% milk. Pierce ECL WB substrate (Thermo Scientific) was added onto the membranes for 2 minutes before film development.

**Primary Antibody**	**Dilution Primary Antibody**	**Protein Size (kDa)**	**Blocking and Antibody Incubation Solution**
Mouse anti-alpha smooth muscle actin (Abcam, ab7817)	1:1000	42 kDa	5% Milk in TBST
Rabbit anti-calponin (Abcam, ab46794)	1:1000	35 kDa	5% Milk in TBST
Rabbit anti-S100A4 (Abcam, ab124805)	1:1000	12 kDa	5% Milk in TBST
Rabbit anti-alpha-tubulin (Abcam, ab15246)	1:1000	52 kDa	5% Milk in TBST
Rabbit anti-GAPDH (Sigma, G9545)	1:500	35 kDa	5% Milk in TBST

Table S2 - Used primary and secondary antibodies for western blotting and corresponding dilutions**.**

2.5 Cytokine Array

Supernatants from 72h coculture experiments were analyzed using the Human Cytokine array C5 (RayBiotech, Inc). Previously blocked membranes were incubated with supernatants for 3.5h, followed by an overnight incubation with the Biotinylated Antibody Cocktail. On the second day, HRP-conjugated Streptavidin antibodies were added for 2h. For chemiluminescence detection, Fusion FX chemiluminescence imaging system was used (Vilber), and finally, signal intensities were analyzed using Fiji software.

2.6 Quantitative assessment of island and network regions in EC images

The ECs visualized by ERG1 and VE-cadherin staining was segmented to island and network regions via texture analysis, highlighting the local features of the brightness pattern.

The following procedure was all carried out using a custom MATLAB script. The horizontal and vertical resolutions of ERG1/VE-cadherin image were first reduced to 10% of the original ones. The image contrast was then tuned using "imadjust" function of MATLAB so that the bottom 1% and the top 1% of the resulting image pixels turned zero and saturate, respectively, followed by conversion to an 8-level grayscale image. Next, an 8 x 8 gray-level co-occurrence matrix (GLCM) was created by counting how often a pixel with grayscale value i occured 3 pixels horizontally and 3 pixels vertically adjacent to a pixel with the value j. The pixels counted in the central elements around the principal diagonal of GLCM (i.e., considerably bright pixels having a neighbor of the similar brightness 3 x 3 pixels away) were categorized to EC island, while those counted in the elements locating around the right-top and the left-bottom corners of the GLCM (i.e., pixels having a considerable contrast to the neighbor 3 x 3 pixels away) were categorized to EC network. The pixels counted in the (1, 1) element (top-left) of the GLCM (i.e., dark pixels having a dark neighbor 3 x 3 pix away) was the background. The obtained binary masks representing island and network regions were finalized by filling gaps spanning smaller than 3 pixels by sequentially applying dilating and eroding morphological operations. The outcomes were used to calculate the coverage of island and network in an arbitrary area.

The GLCM-based analysis captured the difference in the morphological features of the ECs in islands and networks well. It should be, however, noted that the analysis inevitably causes a segmentation error with ECs locally having an irregular contrast or those under inhomogeneous illumination, because it purely focuses on the local feature of the image and does not implement any biological context for segmentation. For quantification, we set several continuous subregions spanning from the periphery to the center of the entire image as a region of interest, keeping the error rate less than 1%. In principle, the GLCM-based analysis detects gaps between networks and holes within an island more strictly than a simple threshold-based area detection.

3. SUPPLEMENTARY VIDEOS

Video S1 – Orbital Shaker. 6-well plate on top of an orbital shaker rotating at 134 rpm. The travelling wave that circulates through the well creating a pulsatile flow can be observed.

Video S2 – High-resolution z-stack video of the IH model at the periphery and center of the well. Low-density coculture with s-SMC (IH-model, prepared as described in **Figure 1A**), after 3 days on the orbital shaker. Videos show a Z-stack acquired through confocal microscope **(A)** at the periphery and **(B)** center of the well. The videos start at the bottom of the well and finish at the top. A 40x water objective was used. Tiles of 3x3 images were stitched together using microscope software (Zen). ECs stained with VE-cadherin antibody by immunostaining (green) and SMC with cell tracker (red). Scale bar 200 µm.

Video S3 – High-resolution z-stack video of corrugated structures in the IH model at periphery of the well. Low-density coculture with s-SMC (IH-model, prepared as described in **Figure 1A**) after 3 days on the orbital shaker. Videos show a z-stack acquired with a Zeiss confocal microscope corresponding to orthogonal views in **(A) Figure 3Ca** and **(B) Figure 3Cb**, respectively. The videos start at the bottom of the well and finish at the top. A 40x water objective was used. Tiles of 3x3 images were stitched together using microscope software (Zen). ECs stained with VE-cadherin antibody by immunostaining (green) and SMC with cell tracker (red). Scale bar 200 µm.

Video S4 – 3D reconstruction of corrugated structure in IH the model. Low-density coculture with s-SMC (IH-model, prepared as described in **Figure 1A**) after 3 days on the orbital shaker. Same sample as in Figure 3C, but image acquired in another location. Video shows a 3D reconstruction of a z-stack acquired with a Zeiss confocal microscope. A 40x water

objective was used. Tiles of 3x3 images were stitched together using microscope software (Zen). Imaris software was used to create animation. ECs stained with VE-cadherin antibody by immunostaining (red) and SMC with cell tracker (green). Scale bar 100 µm.

Video S5 – High-resolution z-stack video of the high-density model at the periphery and center of the well. High-density coculture with s-SMC (high-density model, prepared as described in **Figure 4**) after 3 days on the orbital shaker. Same sample as in **Figure 4A** was used. Videos show a z-stack acquired with a confocal microscope at **(A)** the periphery and **(B)** the center of the well. The videos start at the bottom of the well and finish at the top. A 40x water objective was used. Tiles of 3x3 images were stitched together using microscope software (Zen). ECs stained with VE-cadherin antibody by immunostaining (green) and SMC with cell tracker (red). Scale bar 200 µm.

Video S6– High-resolution z-stack video of the high-density model at the periphery of the well. High-density coculture with s-SMC (high-density model, prepared as described in **Figure 4**) after 3 days on the orbital shaker. Videos show a z-stack acquired with a Zeiss confocal microscope corresponding to the orthogonal view in **Figure 4C**. The video starts at the bottom of the well and finishes at the top. A 40x water objective was used. Tiles of 3x3 images were stitched together using microscope software (Zen). ECs stained with VE-cadherin antibody by immunostaining (green) and SMC with cell tracker (red). Scale bar 200 µm.
